# Paving the way towards an effective treatment for multiple sclerosis: advances in cell therapy

**DOI:** 10.1038/s41423-020-00618-z

**Published:** 2021-05-06

**Authors:** M. J. Mansilla, S. Presas-Rodríguez, A. Teniente-Serra, I. González-Larreategui, B. Quirant-Sánchez, F. Fondelli, N. Djedovic, D. Iwaszkiewicz-Grześ, K. Chwojnicki, Đ. Miljković, P. Trzonkowski, C. Ramo-Tello, E. M. Martínez-Cáceres

**Affiliations:** 1 Division of Immunology, LCMN, Germans Trias i Pujol University Hospital and Research Institute, Barcelona, Spain; 2 Department of Cellular Biology, Physiology and Immunology, Universitat Autònoma de Barcelona, Bellaterra, Spain; 3 Multiple Sclerosis Unit, Department of Neurosciences, Germans Trias i Pujol University Hospital, Barcelona, Spain; 4 Department of Medicine, Universitat Autònoma de Barcelona, Bellaterra, Spain; 5 Department of Immunology, Institute for Biological Research “Siniša Stanković”- National Institute of Republic of Serbia, University of Belgrade, Belgrade, Serbia; 6 Department of Medical Immunology, Medical University of Gdańsk, Gdańsk, Poland; 7 Poltreg S.A., Gdańsk, Poland; 8 Department of Anaesthesiology & Intensive Care, Medical University of Gdańsk, Gdańsk, Poland

**Keywords:** multiple sclerosis, cell-based therapy, tolerance, neuroprotection, autoimmunity, Autoimmunity, Immunosuppression

## Abstract

Multiple sclerosis (MS) is a leading cause of chronic neurological disability in young to middle-aged adults, affecting ~2.5 million people worldwide. Currently, most therapeutics for MS are systemic immunosuppressive or immunomodulatory drugs, but these drugs are unable to halt or reverse the disease and have the potential to cause serious adverse events. Hence, there is an urgent need for the development of next-generation treatments that, alone or in combination, stop the undesired autoimmune response and contribute to the restoration of homeostasis. This review analyzes current MS treatments as well as different cell-based therapies that have been proposed to restore homeostasis in MS patients (tolerogenic dendritic cells, regulatory T cells, mesenchymal stem cells, and vaccination with T cells). Data collected from preclinical studies performed in the experimental autoimmune encephalomyelitis (EAE) model of MS in animals, in vitro cultures of cells from MS patients and the initial results of phase I/II clinical trials are analyzed to better understand which parameters are relevant for obtaining an efficient cell-based therapy for MS.

## Multiple sclerosis

Multiple sclerosis (MS) is a chronic inflammatory and demyelinating disease that affects the central nervous system (CNS) and is characterized by inflammation, multifocal demyelination, axonal loss, and gliosis in both the white and gray matter. MS is a complex disease with considerable clinical and radiological heterogeneity. It was initially classified into four different phenotypes: relapsing-remitting MS (RRMS), secondary-progressive MS (SPMS), primary-progressive MS (PPMS), and relapsing-progressive MS (RPMS).^1^ RRMS (85%) is characterized by acute relapses (acute or subacute episodes of new or increasing neurologic dysfunction in the absence of fever or infection) followed by remission with full or partial recovery. SPMS is defined as progressive clinical worsening over time after an initial relapsing course, with or without acute exacerbations during the progressive course. PPMS, accounting for ~15% of MS, is characterized by clinical progression without relapse from disease onset. The term RPMS is used to describe the progressive accumulation of disability from onset with occasional relapses. This subtype is rarely diagnosed since it overlaps with other phenotypes in terms of its features.

In addition, since 2014, these phenotypes have also included the concept of “disease activity” based on clinical and MRI criteria in an effort to achieve better patient classification.^2^ For MS diagnosis, a combination of clinical, radiological, and laboratory criteria (presence of oligoclonal bands in the cerebrospinal fluid (CSF)) is used. The most recent diagnostic criteria are the 2017 McDonald criteria.^3^

### MS pathogenesis

Currently, the cause of MS remains unknown. In experimental autoimmune encephalomyelitis (EAE), an animal model of MS, myelin-specific T cells are believed to play a crucial role in its pathogenesis.^4^ In fact, the presence of circulating myelin-reactive T cells in MS patients has been extensively reported. However, the specific mechanisms that cause the activation and entrance of these cells into the CNS are still unknown. It has been postulated that a complex interaction between multiple genetic and environmental factors contributes to the dysregulation of peripheral immune homeostasis and the activation of autoreactive T cells.^5^

Several environmental factors, including infectious agents (mainly viruses), tobacco, diet (long-chain fatty acids, salt), gut microbiota, stress, sex hormones, and vitamin D deficiency, have been shown to be related to the triggering and development of disease.^5^ The incidence of MS is higher in women than in men. MS symptoms often improve during late pregnancy, coinciding with high levels of estriol.^6^ In contrast, men are more prone to develop PPMS later in life, correlating with the physiological decline in testosterone with age.^7^

Extensive studies have been performed to understand the genetic contribution to MS, and more than 200 loci that promote a predisposition to MS have been identified, suggesting a complex disease etiology. Nearly all the gene regions identified so far contain genes involved in immune mechanisms. The major *HLA-DRB1*^∗^*1501* locus accounts for 30% of the overall risk.^8,9^ Moreover, emerging evidence indicates that the DNA methylome actively participates in gene × environment interactions, and several studies have shown an aberrant DNA methylome profile develops in MS.^10^

Factors such as the presence of dysfunctional regulatory T cells (Tregs)^11^ or dendritic cells (DCs)^12^ and alterations in cytokine production may facilitate the entry of proinflammatory myelin-specific autoreactive T cells into the CNS (reviewed in^13,14^). In this context, IFN-γ-producing Th17.1 (CCR6^+^CXCR3^+^CCR4^–^) cells have been identified as relevant in disrupting the permeability of the BBB in MS.^15^ Memory B cell precursors and IFN-γ-producing CD8^+^ T cells also express high levels of CCR6, as they can enter the CNS. In addition to chemokine receptors and proinflammatory cytokines, adhesion molecules such as integrin α4β1 (VLA-4), which induces firm adhesion to vascular cell adhesion protein 1 (VCAM-1) on brain endothelial cells, and activated leukocyte cell adhesion molecule enhance the transmigration of pathogenic B and T cell subsets.^16,17^

As an alternative to the immune-mediated cause of MS, it has been postulated that CNS-intrinsic events (for example, CNS viral infection or processes leading to primary neurodegeneration) may trigger disease development, with the infiltration of autoreactive lymphocytes occurring as a secondary phenomenon.^18,19^

The brain and spinal cord are surrounded by the meninges, which provide first-line protection to the CNS. They are constituted by three layers: the dura mater, located directly under the skull or vertebral column; arachnoid mater; and pia mater, in close contact with the CNS parenchyma. The CSF drains through the subarachnoid space, an anatomical gap between the arachnoid mater and pia mater (both known as the leptomeninges). Interestingly, the existence of a rudiment of lymphatic vessels within the meninges was recently described, supporting the existence of a physical connection between the fluids, immune cells, and macromolecules of the CNS and the deep cervical lymph nodes, establishing physical contact between the CNS and the immune system.^19,20^

Most knowledge of MS pathogenesis has been obtained in the EAE model, the animal model of MS. This model is typically induced by either active immunization with myelin-derived proteins or peptides in adjuvant or by passive transfer of activated myelin-specific CD4+ T lymphocytes and reproduces most of the main clinical and histopathological characteristics of MS. Although this model has some limitations, as it is an induced model, it is a powerful tool to investigate MS pathogenesis and potential therapeutic strategies.^21,22^ In EAE, several days before inflammatory cells are detected in the CNS, an influx of peripherally derived immune cells within the meninges occurs.^23^ Myelin antigens that drain from the CNS to the meninges via the CSF are presented by infiltrating or resident antigen-presenting cells (APCs) (perivascular macrophages or resident APCs, e.g., microglial cells).^24–26^ This causes myelin-specific T cell reactivation. Following myelin recognition by autoreactive pathogenic T cells, a complex immune response is produced, facilitated by the entrance of other cell types (B cells, NK cells, macrophages, and innate immune cells) and the production of proinflammatory cytokines and reactive oxygen and nitrogen species (ROS and RNS, respectively); this results in disruption of the blood–brain barrier (BBB) and the entrance of cells into the CNS parenchyma, leading to perivascular inflammation, demyelination, and neuronal damage.^27^

Interestingly, in contrast to EAE, CD8^+^ T cells are found more frequently than CD4^+^ cells in acute and chronic MS lesions. CD8^+^ T cells directly attack oligodendrocytes (via the secretion of granzymes and perforin), causing apoptosis and damaging neurons via the release of cytolytic granules, leading to axonal dissection.^28^ Up to a quarter of CD8^+^ T cells in the active lesions of MS patients can produce IL-17 and are thought to be mucosa-associated invariant T (MAIT) cells. It has been suggested that these CD8^+^ cells, characterized by the expression of a semi-invariant T cell receptor (a dimer of Vα7.2 with Jα12, Jα20, or Jα33), play an important role in disease pathogenesis.^29^

In recent years, the pathogenic role of B cells in MS has been highlighted.^30^ Clonally expanded B cells can be found in the meninges, parenchyma and CSF. B cells produce antibodies intrathecally with an oligoclonal pattern (which can be observed by comparison with serum samples from the same patient). Ectopic lymphoid follicles, which sequester antigens and facilitate B and T cell activation, have been observed proximal to cortical demyelinating lesions in the meninges of MS patients, and their frequency correlates with disease severity.^31–33^ The presence of tertiary lymph follicles suggests that B cell maturation is sustained locally, contributing to the intrathecal synthesis of immunoglobulins.^31,32^ In addition to their roles in antigen presentation to Th1 and Th17 cells and antibody production, B cells secrete proinflammatory cytokines (e.g., IL-6, TNF, granulocyte-macrophage colony-stimulating factor (GM-CSF)), promoting CNS inflammation and demyelination.^34^

There is increasing evidence that innate immune cells (NK, DCs, macrophages, mast cells, and innate lymphoid cells (ILCs)), normal residents of the meninges, are involved in the pathogenesis of MS, affecting both its initiation and progression (reviewed by Brown et al.^33^). Mast cell transcripts that encode mast cell-associated molecules, such as tryptase histamine or FcεR1, have been observed in the demyelinating lesions of MS patients.^35^ They can increase BBB permeability, contributing to the initiation of chronic inflammation.^36^ Moreover, interactions between resident mast cells and autoreactive T cells in the meninges induce caspase-1-dependent IL-1β production by mast cells, activating GM-CSF production by T cells.^37,38^ GM-CSF is an essential growth factor for T cell encephalitogenicity, inducing the recruitment of CCR2^+^ inflammatory monocytes into the CNS.^39–42^ Additionally, TNF expression by mast cells is essential for the early recruitment of neutrophils to the meninges and CNS.^23,36,43^ Neutrophil products, i.e., proteolytic enzymes, including matrix metalloproteinase 9, ROS and structures composed of DNA and proteins called neutrophil extracellular traps, damage the BBB, thus supporting a role for neutrophils in MS pathogenesis.^43–45^

CD45^+^Lin−IL-7Rα^+^ ILCs constitute a heterogeneous group of innate immune cells that have more recently been related to MS pathogenesis: group 1 (ILC1), group 2 (ILC2), and group 3 (ILC3) cells, analogous to Th1, Th2, and Th17 cells, respectively. These cells mostly remain in tissues and exert their effects locally.^46^ Similar to Th cells, ILCs can also exhibit considerable phenotypic plasticity.^46^ Each group of ILCs plays a different role in EAE-related inflammatory responses. ILC2s express ST2, the heterodimeric IL-33 receptor. Upon activation, these cells produce the Th2 cytokines IL4, IL-5, IL-9, and IL-13.^47^ IL-33 induces ST2^+^ ILC2s to produce IL-13 and other Th2-polarizing cytokines, which in turn promote a nonpathogenic Th2-dominated response. In contrast, ILC1s and ILC3s produce IFN-γ, IL-17, GM-CSF, and other cytokines that have been linked to EAE pathogenesis. Interestingly, the lymphoid tissue inducer subset of ILC3s drives ectopic lymphoid follicle formation.^48^

Natural killer (NK) cells have been widely studied in the context of MS and EAE and assigned both pathogenic and protective roles (reviewed in^49,50^). CD56^dim^ cells, the major cytotoxic NK population, can kill oligodendrocytes, astrocytes, and microglia in vitro. In contrast, regulatory CD56^hi^ NK cells produce neurotrophic factors such as brain-derived neurotrophic factor (BDNF) and neurotrophin-3, consistent with a role in neural repair.

Additionally, CNS-resident cells (mainly microglia) are highly sensitive to homeostatic disturbances and can produce neurotoxic inflammatory mediators (cytokines, chemokines, and ROS), promoting and sustaining neurodegeneration (reviewed in^13^). Importantly, astrocytes are key players recruiting lymphocytes and inducing the innate response during the early stage of white matter lesion formation. In contrast, astrocytes can also restrict inflammation through scar formation and trigger neuroprotection and tissue repair.^51^ Regulatory B cells (Bregs), T cells (CD4^+^FoxP3^+^ (Tregs), CD4^+^Tr1^+^ and CD8 Tregs), tolerogenic DCs (tolDCs) and regulatory CD56^hi^ NK cells can regulate effector T cells in the periphery or CNS through different mechanisms. It has been postulated that either a defect in the regulatory function of these cells or the increased resistance of effector T cells to suppressive mechanisms contributes to the pathogenic function of autoreactive T and B cells in MS. Alternatively, the dysfunction of peripheral regulatory cells could be indirectly driven by the dysregulation of tolerogenic APCs (reviewed in^13^).

### Neuropathology of MS

In contrast to the increased inflammation of relapsing forms of MS, neurodegenerative events are more severe in progressive MS. Nevertheless, there is no clear distinction between progressive and relapsing MS; both pathobiologies can occur during the disease.

**Fig. 1:**
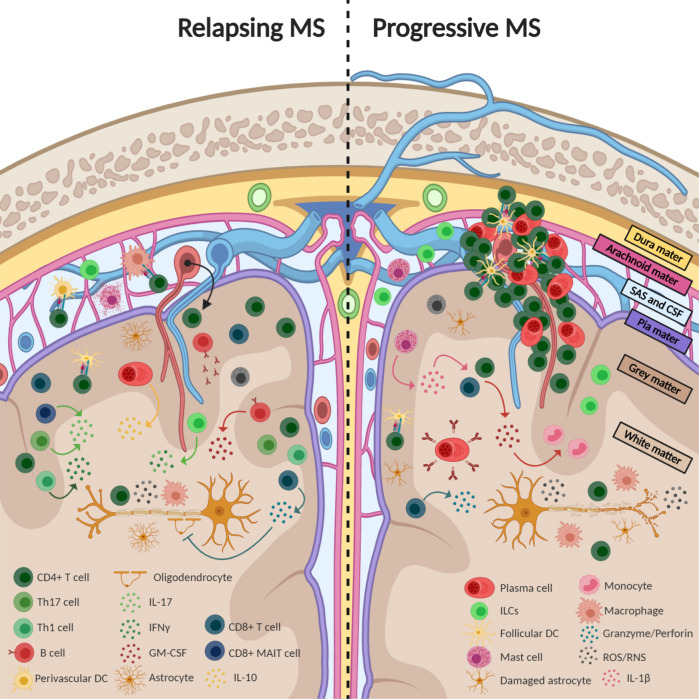
Multiple sclerosis pathogenesis in both relapsing and progressive disease. Scheme representing the major cells and molecules that play a role in the two different stages of MS. The dashed line allows comparison of the differences between relapsing and progressive MS. Arrows indicate release, while inhibitors indicate inhibition. The black arrowhead on a dotted line indicates transmigration. CSF cerebrospinal fluid, DC dendritic cells, GM-CSF granulocyte-macrophage colony-stimulating factor, IFN interferon, IL interleukin, ILCs innate lymphoid cells, MAIT cells mucosal-associated invariant T cells, MS multiple sclerosis, RNS reactive nitrogen species, ROS reactive oxygen species, SAS subarachnoid space, Th T helper

Comparison of PPMS and SPMS reveals some quantitative differences in the presence of focal and active classical white matter lesions and in the global degree of inflammation, which is lower in PPMS than in SPMS.^52^ These differences can be explained by the two types of inflammation in MS patients (Fig. 1). During disease progression, patients can exhibit both types of inflammation, although the composition can be altered during relapses and progressive periods.

During the acute and relapsing phases of the disease, in which leakage of the BBB occurs, the major players are T (CD4^+^ and CD8^+^) and B lymphocytes that attack myelin, leading to demyelination. CD8^+^ cells have the phenotype of tissue-resident memory cells. CD8^+^ T cells proliferate focally and show signs of activation or clonal expansion, indicating local antigen recognition. The pathogenic role of B cells is inferred by the beneficial effects observed after anti-CD20 therapies. However, B cells are thought to play different roles depending on their stage of differentiation or the activity stage of the lesions. Plasmablasts and plasma cells within MS lesions express high levels of IL-10,^53^ suggesting that they may ameliorate inflammation.

Inflammatory infiltrates may lead to focal areas of primary demyelination with variable axonal injury, which is mainly carried out by activated microglia and macrophages. Antibodies that recognize oligodendrocytes or astroglia may contribute to MS pathogenesis at this stage. After the initial autoimmune attack, lymphoid cells in the parenchyma undergo apoptosis, and macrophages and microglia can switch to an anti-inflammatory/reparative phenotype.

In the progressive phases of MS, the second pattern of inflammation predominates. Leakage of the BBB is less pronounced, and T and B lymphocytes slowly accumulate in the connective tissue spaces of the brain and spinal cord, affecting the meninges and periventricular spaces. Infiltrating cells form focal aggregates resembling tertiary lymph follicles. CD8^+^ cells have the phenotype of tissue-resident memory cells with focal activation. Most cells of the B cell lineage in chronic lesions are plasmablasts and plasma cells. Tissue injury may at least be partly mediated by microglia and macrophage activation, oxidative injury and mitochondrial damage. This inflammation is associated with the expansion of pre-existing lesions, as well as diffuse neurodegeneration in normal-appearing white or gray matter. Interestingly, it has been found that this second type of inflammation is already present in the early stages of MS, after which it increases gradually with disease duration and patient age.^52^ The inflammation induced by peripheral immune cell infiltration and CNS-resident innate immune cells may contribute to acceleration of the aging processes in the CNS, followed by pronounced progressive neurodegenerative decline (reviewed in^13^).

## Cell-based tolerogenic therapies

**Fig. 2:**
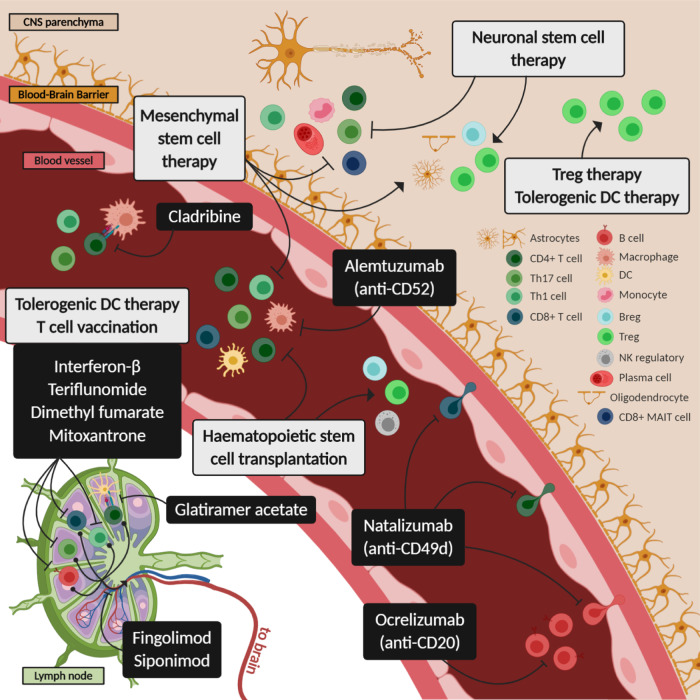
Proposed mechanisms of action of approved treatments for multiple sclerosis and cell-based therapies. Representation of the mechanisms of action of current treatments (black boxes) and cell-based therapies (gray boxes). Arrows indicate induction, while inhibition symbols indicate inhibition. Breg regulatory B cells, CNS central nervous system, DC dendritic cells, MAIT cell mucosal-associated invariant T cells, NK natural killer cells, Th T helper, Treg regulatory T cells

Targeting the fundamental cause of autoimmunity, i.e., the loss of tolerance to self-antigens, will provide the next steps forward to avoid the general immunosuppression induced by current treatments. Accumulating knowledge on the mechanisms of immune tolerance and activation has led to the development of tolerance-inducing cellular therapies with the specific objective of limited unwanted immune reactions over the long term (reviewed in^54^). Phase I clinical trials using Tregs, mesenchymal stem cells (MSCs), or tolerogenic antigen-presenting cells, such as tolDCs and regulatory macrophages, for the treatment of autoimmune diseases (ADs) and prevention of transplant rejection have expanded in recent years. The results have confirmed so far that these cellular therapies are safe, with no relevant side effects, and well tolerated by patients.^54^ In this context, several cellular products have been developed for MS (Fig. 2), and phase I clinical trials are currently ongoing or finished. To advance the use of these therapies in the clinic, we should analyze the results and address remaining challenges, such as the optimal dose, administration route, frequency of administration, antigen specificity, and biomarkers of clinical and biological response, to design the next generation of clinical trials.

### Tolerogenic dendritic cells

DCs are key players in controlling the immune response. They are highly efficient APCs that are able to activate the immunogenic T cell response and suppress it by inducing regulatory mechanisms.^55^ TolDCs are defined as semimature DCs with an intermediate phenotype between the phenotypes of immature (iDCs) and mature DCs (mDCs). It is not clear whether tolDCs constitute a different DC subset by themselves. TolDCs are characterized by one or more of these features: the expression of low levels of costimulatory molecules (i.e., CD80, CD86, and CD40) and MHC class II, a reduced capacity to produce proinflammatory cytokines, upregulated expression of inhibitory molecules such as PDL1, ILT3 and ILT4 and secretion of immunoregulatory cytokines and mediators (IL-10, TGF-β, IDO, heme oxygenase-1 or FasL).^56,57^ Because of the semimature or mature resistance phenotype of tolDCs, the proper activation of T cells by costimulatory molecules and proinflammatory cytokines following antigen recognition is limited. Under these conditions, instead of inducing the activation and clonal expansion of T cells, tolDCs promote T cell hyporesponsiveness that is mainly mediated by the induction of T cell anergy, T cell depletion triggered by apoptosis induction or the induction of Treg differentiation (reviewed in^58,59^).

Importantly, tolDCs can be generated ex vivo from autologous human peripheral blood monocytes. Indeed, over the last 20 years, a wide variety of protocols describing the induction of tolDCs with several stimuli, such as anti-inflammatory cytokines (IL-10 and TGF-β), pharmacological agents and immunosuppressant compounds (rapamycin, different corticosteroids, vitamin D3, aspirin, mitomycin C, and the NF-κB inhibitor BAY11-7082), and other strategies, such as genetic engineering for the selective repression or induction of key molecules and pathways, among many others, have emerged (reviewed in^54,58^). Most of these protocols share several features, such as the differentiation of monocytes in the presence of GM-CSF and IL-4, as well as the addition of a maturation stimulus (which usually consists of different combinations of LPS, monophosphoryl lipid A, TNF, IL-1β, prostaglandin E2 (PGE2) and IL-6), to maintain tolDCs in an activation-resistant state, as this is an absolute requirement for tolDC therapy.

Analysis of preclinical data for the evaluation of tolDC treatment in EAE, as well as in vitro studies on peripheral blood leukocytes from MS patients (Table 1), is crucial to better understand the requirements and characteristics for the design of efficient tolDC products for MS patients.

**Table 1:** Tolerogenic DCs therapies in EAE and in vitro studies using MS patients tolDCs

Tolerogenic DC therapies in EAE^a^										
Tolerogenic agent	EAE model	Groups of treatment	Number of administrations	Type of treatment^b^	Route	Number of tolDCs	Clinical outcome	Mechanism of action	Observations	Ref
K313	C57BL/6 mice immunized with MOG_35–55_	MOG-K313-DC, unpulsed K313-DC and MOG-VitD3-tolDCs (control group)	3 administrations (every 3 days) at days 10, 13, and 16 p.i.	Early therapeutic	i.v.	1 × 10^6^ cells	Clinical symptoms amelioration (similar to MOG-VitD3-tolDCs group)	Strong reduction of leukocyte infiltration and demyelination Reduction of Th1 and Th17 cells and increase of Treg compared to untreated group	BMDCs treated with K313 inhibit antigen-specific CD4+ T cells	^60^
VitD3	C57BL/6 mice immunized with MOG_35–55_	MOG mRNA electroporated-VitD3-tolDCs, MOG-VitD3-tolDCs, unpulsed VitD3-tolDCs and PBS	3 administrations (every 4 days) at days 13, 17, and 21 p.i.	Therapeutic	i.v.	1 × 10^6^ cells	Reduced disease severity with both electroporated and pulsed antigen-specific VitD3-tolDCs	Decrease of number of spinal cord lesions (MRI) Decreased MOG-reactive T cells in the spleen and lymph nodes and reduction of proinflammatory cytokines secretion (IL-17, IFN-γ, TNF-α, GM-CSF)		^61^
	C57BL/6 mice immunized with MOG_35–55_	MOG-VitD3-tolDCs, MOG-DC and PBS	3 administrations (every 3 days) at days 10, 13, and 16 p.i.	Early therapeutic	i.v.	0.8 × 10^6^ cells	Delay of disease onset Reduction of EAE severity	Decreased Th1 and Th17 cell infiltration into the CNS Enhanced % of Treg, CD4^+^ IL10^+^ T cells and Breg in spleen and/or lymph nodes	No maturated DC	^62^
	C57BL/6 mice immunized with MOG_35–55_	MOG and unpulsed engineered tolDCs overespressing VitD3 and untreated EAE	1 single administration at day 10 p.i. or 3 administrations (every 7 days) at days 10, 17, and 24 p.i.	Early therapeutic	i.v. (1 dose) s.c. (3 doses)	1 × 10^6^ cells	Reduced disease severity with antigen-specific manner	Less inflammatory foci and demyelination Increase of Th2, Tr1, and FoxP3^+^ Treg cells Higher secretion of IL-4 and IL-10 compared to control groups Clinical amelioration related with FoxP3^+^ Treg induction	DC engineered to overexpress 25-hydroxyvitamin D 1α-hydroxylase (self-tolerized) and release 1,25-dihydoxyvitamin D	^63^
	C57BL/6 mice immunized with MOG_40–55_	Cryopreserved MOG-VitD3-tolDCs, unpulsed VitD3-tolDCs and PBS	For short-term treatment (30 days), 3 administrations (every 4 days: 15, 19, and 23 p.i.) For long-term treatment (74 days), 3 initial administrations (every 4 days: 14, 18, and 22 p.i.) + extra-administrations when the mean clinical score of the MOG-VitD3-tolDCs group increased	Therapeutic	i.v.	1 × 10^6^ cells	Reduced disease severity following short and long-term antigen-specific treatment with antigen-specific VitD3-tolDCs	Short-term treatment: Inhibition of MOG_40–55_ T cell reactivity and increased proportion of FoxP3^+^ Treg Long-term treatment: Inhibition of MOG_40–55_ T cell reactivity; increase of Breg and activated NKT cells; and decreased proportion and activation of NK cells	The long term-treatment with cryopreserved tolDCs-VitD3-MOG was well tolerated, highly effective and exhibited a prolonged clinical benefit after each administration.	^64^
	C57BL/6 mice immunized with MOG_40–55_	MOG-VitD3-tolDCs, unpulsed VitD3-tolDCs and PBS	2 administrations for prophylactic (−2, 5 p.i.) and late-prophylactic (5, 9 p.i.) approaches and 3 administrations (every 4 days: 15, 19, and 23 p.i.) for therapeutic	Prophylactic, late-prophylactic and therapeutic	i.v.	1 × 10^6^ cells	Antigen-specific VitD3-tolDCs showed reduced EAE incidence (prophylactic treatment) and reduced disease severity (late-prophylactic and therapeutic treatment)	Induction of FoxP3^+^ Treg and IL-10 secretion Inhibition of MOG_40-55_-specific T cells reactivity	Biodistribution analysis showed that although tolDCs reached CNS following i.v. injection, tolDCs accumulated in spleen after 48 h and remained there at day 14 p.i.	^65^
Chloroquine	C57BL/6 mice immunized with MOG_35–__55_	MOG-CQ-BMDC, MOG-PBS-BMDC and untreated EAE	1 single administration at day 10 p.i.	Late-prophylactic	i.v.	1.5 × 10^6^ cells	Reduction of EAE severity Suppression of Th17 infiltration into the CNS	Inhibition of T cell proliferation and reduction of differentiation and infiltration of Th17 cells mediated by STAT1 signaling Increase of IL10 secretion and reduction of IFN-γ levels Induction of FoxP3+ Treg	STAT1^−/−^ mice were also used in the experiments and treated with MOG-CQ-BMDC or MOG-PBS-BMDC	^66^
Tofacitinib	C57BL/6 mice immunized with MOG_35–__55_	MOG-Tofa-DC, unpulsed-Tofa-DC PBS, and non-immunized	3 administrations (every 4 days) at days 7, 11, and 15 p.i.	Early therapeutic	i.v.	1 × 10^6^ cells	Reduction of EAE severity in mice receiving antigen-specific Tofa-DC	Reduced leukocyte infiltration and less extensive demyelination into the CNS Decreased % of Th1 and Th17 cells and enhanced % of CD25 + FoxP3+ Treg in the spleen	Frequencies of Th17 and Th1 cells correlated positively with the clinical score and correlated negatively with the frequency of Treg	^67^
BD750	C57BL/6 mice immunized with MOG_35–__55_	MOG-BD750-tolDCs, npulsed BD750-tolDCs, PBS and non-immunized	3 administrations (every 4 days) Prophylactic approach: days −2, 2, and 6 p.i. Early therapeutic approach: days 7, 11 and 15 p.i. Late-therapeutic approach: days 19, 23, and 27 p.i.	Prophylactic, early therapeutic and late-therapeutic	i.v.	1 × 10^6^ cells	Early therapeutic treatment: Delay on disease onset and reduced EAE severity with MOG-BD750-tolDCs Late-therapeutic treatment: no clinical improvement	Early therapeutic: Reduced inflammatory infiltrates and demyelination in the CNS Reduced frequency of Th1 and Th17 cells and increased % of FoxP3^+^ Treg in the spleen	Early therapeutic treatment using only 1 or 2 administrations of MOG-BD750-tolDCs (at day 7 p.i. or days 7 and 11 p.i.) showed no clinical efficacy	^68^
IL-35	C57BL/6 receiving 5·10^6^ MOG_35–__55_-specific T cells (Passive EAE)	MOG loaded and unpulsed tumor DC cell line modified to express IL-35	1 single administration 1 day after passive EAE induction (prophylactic) and 2 administrations at day 6 and 8 post passive EAE induction (late-prophylactic)	Prophylactic and Late-prophylactic	i.v.	2.5 × 10^6^ cells	Reduction of EAE symptoms	Impairment of T cell activation and proliferation		^69^
LPS	C57BL/6 mice immunized with MOG_35–__55_	MOG-LPS-DC, MOG-DC and unpulsed DC	3 administrations (every 4 days) at days 11, 14, and 17 p.i.	Early therapeutic	i.v.	0.3 × 10^6^ cells	Antigen-specific abrogation of EAE development	LPS-treated BMDC increase the frequency of Treg (CD4+ CD25+ FoxP3+ GITR+) that are CD127+ 3G11− and inhibit those CD127 + 3G11 + Treg		^70^
Apoptotic thymocytes	C57BL/6 mice immunized with MOG_35–__55_	Splenic DC primed with irradiated (apoptotic) or fresh T cells and pulsed with or without MOG	3 administrations (every 4 days) at days 11, 14, and 17 p.i.	Early therapeutic	i.v.	0.3 × 10^6^ cells	Antigen-specific abrogation of EAE development	Inhibition of central and effector memory CD4+ T cell development Reduction of IFNγ production	Immune tolerance induced by apoptotic T cell-treated DC is mainly related to CD4+ effector memory T cells reduction	^71^

In vitro studies using tolDCs from MS patients										
Tolerogenic agent	Phenotype		Functionality	Stability	Comparison with HD tolDCs		Mechanism of action		Observations	Ref
VitD3	Reduced expression of CD86, CD83 and HLA-DR molecules Production of higher levels of IL-10 (although not statistically significant) and low levels of IL12p70 compared to mDC		Reduced ability to stimulate T cell allogeneic proliferation Decrease secretion of TNF-α, IL-6, and IFN-γ Induction of antigen-specific T cell hyporesponsiveness	Stable phenotype and functionality LPS stimulation did not induce IL-23 secretion but small amounts of IL-10 were still detectable	Equal efficiency to generate VitD3-tolDCs from MS patients and HD Low level expression of CD83 in mDC from MS patients compared with HD		Not provided		Set up of myelin peptide-tolDCs loading	^82^
	Decreased expression of CD86, CD80, CD83, and HLA-DR molecules		Reduced allogeneic and antigen-specific T cell proliferation Impaired secretion of proinflammatory cytokines	No tested on fresh VitD3-tolDCs Cryopreserved VitD3-tolDCs remained phenotypically and functionally stables following LPS stimulation	VitD3-tolDCs and mDC from MS patients secreted higher levels of IL-1β, IL-6 and TNF-α compared to HD counterparts		Unknown No induction of neither FoxP3^+^ Treg nor IL-10 and/or TGF-β producing cells		Cryopreserved VitD3-tolDCs were studied: They retained phenotypical and functional characteristics and remain stable following proinflammatory stimulus	^75^
	Reduced expression of CD83, CD86, and HLA-DR molecules		Inhibition of allogenic proliferation and increase secretion of IL-10	Non evaluated	VitD3-tolDCs from HD also exhibited increase secretion of TGF-β		No provided		*MUCL1* and *MAP7* were reported as biomarkers of both HD and MS patients VitD3-tolDCs	^87^
Ethyl pyruvate (EP)	Decreased expression of CD86, CD83, and HLA-DR molecules		Inhibition of allogenic T cell proliferation with reduction of IFN-γ. No increase of IL-10 levels was observed	Non evaluated	No differences in neither phenotype nor allogenic proliferation inhibition of HD and MS patients		No provided		Similar phenotype and functionality of EP-tolDCs and VitD3-tolDCs	^86^

*CNS* central nervous system, *CQ* chloroquine, *DC* dendritic cells, *EP* ethyl pyruvate, *HD* healthy donors, *i.v.* intravenous, *MAP7* microtubule-associated protein 7, *MOG* myelin oligodendrocyte glycoprotein, *MRI* magnetic resonance imaging, *MS* multiple sclerosis, *MUCL1* mucin-like 1, *Tofa* tofacitinib, *VitD3* vitamin D3

^a^From 2015 to 2020

^b^Prophylactic treatment: treatment before or at the moment of EAE induction; Late-prophylactic treatment: treatment after EAE induction but prior to clinical onset; early therapeutic treatment: initiation of treatment when mice showed first clinical symptoms; therapeutic treatment: first dose administrated to mice with established clinical signs; late-therapeutic: treatment in mice with high degree of paralysis

#### Antigen specificity

Several studies in the EAE model of MS have demonstrated that clinical efficacy of tolDCs is achieved only when the cells are loaded with disease-related autoantigens^60–71^ (Table 1). Although the etiology of MS is unknown, it is widely accepted that it is an autoimmune-mediated disease directed against several myelin proteins, such as MBP, proteolipid protein (PLP), and myelin oligodendrocyte glycoprotein (MOG). Consequently, when thinking about the translation of tolDC therapy to humans, the loading of a single antigen on tolDCs is unlikely to be sufficiently effective. Moreover, due to the epitope spreading phenomenon, the same patient may also exhibit extended reactivity to additional myelin epitopes following disease progression. Therefore, in tolDC phase I clinical trials for MS patients, a cocktail of myelin peptides containing the most relevant autoreactive MOG, PLP, and MBP peptides seems to be a better strategy.^72,73^ In fact, the three phase I clinical trials of peptide-loaded tolDCs in MS have employed a similar approach using a pool of myelin peptides (NCT02283671, NCT02903537, and NCT02618902) (Table 2).

**Table 2:** Clinical trials using tolDCs treatment in MS patients

Type of tolDCs	NCT number	Purpose	Number of patients	Phase	Status	Antigen	Dose	Number, route, and frequency of administrations	Observations	Ref
Dexamethasone	NCT02283671 TolDec-EM-NMO	—Evaluation of safety and tolerability of dexa-tolDCs treatment —Analysis of changes in the immunological profile	8 MS patients and 4 NMO patients	I	Completed (2019)	Pool of 7 myelin peptides^a^ of MS + AQP463-76 for NMO	Dose-escalation study with a total of 50, 150 and 300 × 10^6^ Dexa-tolDCs	A total of 3 i.v. doses every 2 weeks (week 0, 2, and 4)	Administration of fresh antigen-specific tolDCs The treatment was safe and well tolerated Increase secretion of IL-10 after myelin peptides stimulation at week 12 vs baseline Decrease of memory CD8 + T cells and NK cells by week 12 vs baseline	^74^
Autologous VitD3-tolDCs	NCT02903537 TOLERVIT-MS: Tolerance-Induction with Dendritic Cells Treated with Vitamin-D3 and Loaded with Myelin Peptides, in Multiple Sclerosis Patients	—To determine the safety and tolerability of the intranodal administration of autologous tolDCs-VitD3 pulsed with myelin peptides in multiple sclerosis patients —To select the most appropriate regime for the development of future therapeutic trials —To evaluate the preliminary proof of concept by clinical and/or radiological activity and immunological markers —To test the effect of the selected VitD3-tolDCs doses combined with IFN-β (*n* = 3)	12active MS patients	I	Recruiting	Pool of 7 myelin peptides^a^	Dose-escalation study using 5, 10, and 15 × 10^6^ VitD3-tolDCs/injection Additional cohort: the highest dose of VitD3-tolDCs well-tolerated + IFN-β treatment	A total of 6 intranodal doses (cervical lymph nodes): the first 4 administrations every 2 weeks + the last 2 administrations every 4 weeks (week 0, 2, 4, 6, 10, and 14)	Administration of cryopreserved antigen-specific tolDCs	On going
	NCT02618902 MS-tolDCs: A “Negative” Dendritic Cell-based Vaccine for the Treatment of Multiple Sclerosis: a First-in-human Clinical Trial	—To determine the safety and tolerability of the intranodal administration of autologous tolDCs-VitD3 pulsed with myelin peptides in multiple sclerosis patients —To select the most appropriate regime for the development of future therapeutic trials —To evaluate the preliminary proof of concept by clinical and/or radiological activity and immunological markers	9 active MS patients	I	Recruiting	Pool of 7 myelin peptides^a^	Dose-escalation study using 5, 10, and 15 × 10^6^ VitD3-tolDCs/injection	A total of 6 intradermal doses (in the subclavicular region): the first 4 administrations every 2 weeks + the last 2 administrations every 4 weeks (week 0, 2, 4, 6, 10, and 14)	Administration of cryopreserved antigen-specific tolDCs	On going

*AQP* aquaporine, *Dexa* dexamethasone, *i.v.* intravenous, *NK* natural killer, *NMO* neuromyelitis optica, *VitD3* vitamin D3

^a^MOG_1–__20_, MOG_35–__55_, MBP_13–__32_, MBP_83–__99_, MBP_111–__129_, MBP_146–__170_, and PLP_139–__154_^100,101^

#### Dose, timing, and frequency

Although studies in animal models are extremely useful, extrapolation of the dose, timing, and frequency of administration to patients is not straightforward. From publications using different tolDC types to treat EAE, i.v. administration of a million antigen-specific tolDCs has demonstrated promising results in reducing EAE severity (Table 1). However, extrapolation of the dose from these studies is not feasible. The monocyte-derived tolDCs recovered after the in vitro differentiation process number a maximum of several million, depending on the patient. Consequently, thus far, direct extrapolation of tolDC doses from EAE studies is not feasible. In fact, in a phase I trial of i.v. dexamethasone-tolDC (dexa-tolDC) administration, a technical limitation was reported related to reaching 300 × 10^6^ cells.^74^ Since cells administered intravenously exhibit a wide biodistribution of cells (meaning that a large number of tolDCs would be necessary to reach the secondary lymphoid organs), other routes of administration are currently being tested by our group and others (Table 2). This issue will be discussed below.

As shown in Table 1, many of the EAE studies analyzed the efficacy of tolDCs before the onset of clinical symptoms (prophylactic or late prophylactic administration). In contrast, other authors have attempted to reproduce the real-world situation of MS patients for therapeutic administration.^61,66,67,70^ From those studies, antigen-specific VitD3-tolDCs demonstrated a remarkable therapeutic effect.^61,66,67^

Focusing on therapeutic studies in EAE, the administration of at least 3 doses of antigen-specific tolDCs treated with VitD3 or BD750 (a JAK3/STAT5 inhibitor) in the early stage of disease abrogated disease progression.^61,66,67,70^ Considering translation to MS, the use of multiple tolDC injections seems optimal. However, this would imply repeated production of tolDC batches (which would require several rounds of leukapheresis and in vitro differentiation under good manufacturing practice conditions). Hence, the use of cryopreserved cells is a feasible option.^66,75^ In fact, at least for VitD3-tolDCs, both human and murine cryopreserved cells retain phenotypical and functional tolerogenic characteristics. Importantly, therapeutic administration of cryopreserved MOG_40–55_-VitD3-tolDCs in EAE mice showed clinical efficacy, reducing T cell autoreactivity and triggering the generation of FoxP3^+^ Tregs in vivo.^66^ All these data were comparable with those obtained using fresh cells.^67^ To go one step further, we explored the effect of long-term treatment with cryopreserved MOG_40–55_-VitD3-tolDCs in EAE. Following 3 therapeutic administrations of MOG_40–55_-tolDCs every 4 days, additional doses were administered when the mean clinical score of the group increased. Strikingly, we observed that periods of long-lasting clinical stability increased progressively. Immunological examination of mice revealed increased proportions of Bregs and activated NKT cells as well as a reduction in immunogenic NK cells in the spleens of treated mice, which might explain (at least in part) the beneficial effect of MOG_40–55_-tolDCs.^66^

The effect of tolDCs in the chronic phase of EAE has been analyzed using MOG_35–55_-BD750-tolDCs. Unfortunately, no clinical benefits were observed even though 3 doses of MOG_35–55_-tolDCs were administered every 4 days.^70^ Although more studies are needed, these results suggest that tolDC therapy should be used in the first stages of the disease.

#### Route of administration

The safety of i.v., intraperitoneal (i.p.), intradermal (i.d.), intranodal (i.n.) and even intra-articular routes of human tolDC administration has been demonstrated in different phase I clinical trials.^74,76–79^ The route of administration is crucial due to two important issues. On the one hand, the treatment must promote tolerogenic in vivo effects. In this context, i.v. administration is considered the most tolerogenic route of administration, showing tolerogenic effects superior to those of i.d. administration in nonhuman primates.^80^ On the other hand, the selected route of administration must allow tolDCs to reach the draining lymph nodes or inflamed tissues. In this regard, either i.d. administration near the draining lymph nodes or direct i.n. tolDC injection could be an alternative to i.v. administration. Interestingly, two coordinated phase I clinical trials using VitD3-tolDCs will compare these two routes of administration^81^ (Table 2).

#### Mechanism of action

The mechanism of action of several types of tolDCs has been analyzed.^54^ Most tolDCs impair T cell alloproliferation in in vitro cultures. Moreover, they also act through different pathways. Dexa-tolDCs produce IL-10, tolDCs generated in the presence of exogenous IL-10 (DC-10) induce IL-10-producing Tregs (Tr1 cells), and VitD3-tolDCs induce T cell hyporesponsiveness in an antigen-specific manner without affecting the ability of other T cells to respond to unrelated antigens.^75,82^ In addition, a transcriptomic analysis of autologous CD4^+^ T-cells primed with antigen-specific VitD3-tolDCs revealed profound genetic downregulation, mainly affecting factors related to the cell cycle and proinflammatory immune response processes.^83^

Dexa+VitD3-tolDCs regulate CD4^+^ T cell cytokine production in RA patients in a TGF‐β1‐dependent manner.^84^ Additionally, TGF-β secreted by tolDCs is an important immunoregulatory mediator involved in the induction of Tregs. Interestingly, tolDCs differentiated in the presence of low doses of GM-CSF and in the absence of IL-4, referred to as autologous tolerogenic dendritic cells (ATDCs), have the capacity to reduce T cell proliferation via a novel mechanism involving lactate secretion.^85^ Lactate secreted by ATDCs exerts its immunosuppressive effect by downregulating T cell glycolysis. Currently, the specific mechanisms triggered by different tolDCs in vivo remain elusive. Inhibition of antigen-specific T cell proliferation, increases in FoxP3^+^ Treg numbers, decreases in proinflammatory Th17 and Th1 cell numbers in both the periphery (spleen and lymph nodes) and CNS, and increased levels of IL-10 have been described^60–65,67–70,75,82,84,86,87^ (Table 1). Interestingly, an increased frequency of Bregs was found after MOG-VitD3-tolDC therapy in EAE,^64,66^ similar to the results of the first phase I clinical trial with genetically modified tolDCs conducted by Giannoukakis et al. in patients with type I diabetes.^77^ In MS, an increase in IL-10 production by PBMCs isolated from treated patients was described.^74^ In addition, tolDCs can induce the secretion of indoleamine 2,3-dioxygenase (IDO), a potent regulatory enzyme that catalyzes the degradation of tryptophan required for T cell proliferation.^88^ Altogether, these data indicate that tolDC therapy can trigger a complex tolerogenic immune cascade, with anergy or elimination of pathogenic Th1/Th17 cells and induction of regulatory cells (FoxP3^+^ Tregs, Tr1 cells, and Bregs), that can reduce EAE severity, even in mice with established clinical signs of paralysis.

#### Phenotype, function, and stability

Currently, no common biomarkers of tolerogenic function in different types of monocyte-derived tolDCs have been identified.^89^ Thus, their phenotypic, as well as functional, characterization requires comparison with mDCs generated in parallel to certify their correct production before administration to patients. In addition, the stability of tolDCs is crucial, and stability can be analyzed in vitro in tolDCs exposed to a proinflammatory milieu to ensure that there is no conversion of tolerogenicity to immunogenicity.^90^ No data regarding the stability of these cells in vivo after their administration to patients have been obtained so far. In this context, it is expected that the use of cell trackers will provide relevant information to better understand the in vivo mechanism of action of tolDCs.^91^

#### Clinical trials in MS

To the best of our knowledge, a total of three phase I clinical trials using tolDCs for the treatment of MS patients are ongoing or have recently finished (Table 2). In one dose-escalation phase Ib clinical trial, patients with MS (*n* = 8) and neuromyelitis optica (*n* = 4) received 3 i.v. injections (50, 100, 150, or 300 × 10^6^ tolDCs in total) as three independent doses administered every 2 weeks. However, the last group did not receive the planned doses due to a technical limitation in obtaining the required number of cells. Clinically, the treatment was safe and well tolerated.^74^

Two coordinated phase I clinical trials in MS patients treated with autologous VitD3-tolDCs loaded with myelin peptides are currently ongoing simultaneously in Belgium and Spain in the context of the European H2020 framework.^81^ Both studies tested the safety and tolerability of autologous peptide-loaded VitD3-tolDCs in a dose-escalation study using 5, 10, and 15 × 10^6^ VitD3-tolDCs/administration, with the first four of six independent doses administered every 2 weeks and the last two administered every 4 weeks. In addition, exhaustive immunomonitoring is being performed (Table 2).

### Regulatory T cells

Since Treg induction is one of the most relevant and consistent mechanisms to achieve immunoregulation, cell therapy administering Treg cells is one of the most promising strategies that has been extensively investigated worldwide.^92^

#### Mechanism of action

Tregs in the body comprise the naturally occurring/thymic (tTreg) and induced/peripheral (pTreg) compartments. The latter compartment is further divided into several subsets, with Tr1 cells and Th3 cells being the main representatives. While tTregs are specifically designed to regulate the immune response from the progenitor stage in the thymus, pTregs are generated via the conversion of conventional CD4^+^ T cells in the periphery during the immune response.^93^ Both subsets are efficient regulators of the immune response, but their origins suggest somewhat different activities. Thymic Tregs are anergized towards self-antigens in the thymus. These cells migrate to a site of inflammation and the local lymphoid tissue surrounding it and limit the immune response when self-antigens are sensed. In this way, tTregs protect the body from possible autoreactivity. This suppression is very precise, occurring mainly locally via cell-to-cell interactions with other cells that take part in the immune response. The main receptor of tTregs is CTLA-4 (cytotoxic T lymphocyte antigen 4, CD152), which links to receptors from the B7 family on APCs and limits the presentation of autoantigens. Surface TGF-β and LAG-3 are involved in the suppression of NK cells, and PD-1/PD-L coupling is involved in the suppression of T cells and B cells. High expression of CD25 (IL2Rα subunit) allows tTregs to preferentially take up the majority of available IL-2, which triggers apoptosis of overactivated effector T cells (conventional T cells, Tconvs) in the surrounding microenvironment. This suppression is not limited to self-antigens, as the interaction with APCs can result in so-called “linked suppression”. Specifically, when APCs present tolerized autoantigens with some alloantigens, tTregs interacting with APCs can also impose tolerance towards the alloantigen. This is an important advantage of the polyclonal preparation of tTregs used in cell therapy. The fact that pTregs arise in the periphery during immune responses, mainly from naïve CD4^+^ T cells, implies that they are specific to the antigen that triggers the immune response. The main regulatory mechanism of pTregs involves the secretion of suppressive factors such as IL-10, produced mainly by Tr1 cells, or TGF-β, produced by Th3 cells.^92,94^ Currently, tTregs are the main subset used in the clinic, but a limited number of trials using Tr1 cells have also been conducted.

#### Phenotype, function, and stability

Expression of the transcription factor FoxP3 is currently the main marker of Tregs.^93^ CD127 (the IL7 receptor) is negatively correlated with FoxP3.^11,95^ Importantly, mutations that render FoxP3 inactive in humans are responsible for immune dysregulation, polyendocrinopathy, enteropathy, and X-linked inheritance (IPEX). Nevertheless, the phenotype should always be confirmed with the suppressive function. Throughout their development from the progenitor stage to the immune response, tTregs undergo many changes that affect their activity. For example, there is a developmental link between Th17 cells and tTregs, which implies the plasticity of cells of these phenotypes.^96^ This is important for the stability of Tregs not only during in vitro manufacturing but also when tTregs are expected to function in the body. The stability of Tregs is also substantially affected by epigenetic changes in mature cells. For example, methylation of the Treg-specific demethylated region within the *foxp3* gene significantly impairs suppressive function.^97^ Additionally, the inflammatory cytokine milieu can counteract the suppressive effects of tTregs, which should be considered when therapies with tTregs are designed. For example, TNF secreted by inflammatory cells can abrogate the suppressive effects of tTregs.^98^ This fragile phenotype should be taken into account during the manufacturing of Tregs for clinical applications, since trivial factors such as the time or temperature of expansion might affect the final activity of the cellular product.^99,100^

#### Therapy with Tregs in EAE

While animal studies in many cases assume very early intervention, when there is little or no damage to the CNS, therapy in humans usually starts when the pathology is very advanced, as the first symptoms often occur only then. Likewise, the burden of inflammation and the location of the inflammatory process may be different between an animal model and humans in a trial, and this needs to be considered when translating the results into the clinic. One variable to consider is the time when the treatment is administered. In this context, analysis of different studies on Tregs shows that their administration in EAE was performed at different times: prophylactically (before or with immunization), late prophylactically (before the first clinical signs appeared), therapeutically (with the first clinical signs), and late therapeutically (5–8 days after the initial clinical signs). Details of the studies are given in Table 3. Here, we will focus on studies in which Tregs are administered therapeutically, as they more closely resemble the potential application of Tregs in MS.

**Table 3:** CD4^+^CD25^+^FoxP3^+^ Tregs in EAE treatment

EAE	N^o^ of Tregs	Administration^a^	Modification	Propagation	Phenotype	Effect on EAE	Ref
PLP_178–191_ SJL/J MBP_87–99_ SJL/J MOG_35–55_ (SJL/J x C57BL/6) F1	1 × 10^6^	Prophylactic i.v. 1 d.b.i.	5B6 Tregs	PLP 30 µg/ml	CTLA4^+^ IL-10	Amelioration	^107^
PLP_139–151_ SJL/J	1 × 10^5^	Prophylactic i.v. 2 d.b.i.	No	No	LAP+, CTLA4+, GITR+, ICOS+, PD1+, OX40+, CD103+, Tim3+ TGF-β	Amelioration	^109^
MBP Ac1-9 C57BL/6	1 × 10^5^	Prophylactic i.p. 0 d.p.i.	CRP-MBP mice	No	n.a.	Prevention	^110^
MBP Ac1-9 B10.PL PLP_139–151_ (B10.PL x SJL) F1	1 × 10^5^ 3 × 10^5^ 4–5 × 10^5^ 1 × 10^6^	Prophylactic i.v. 0 d.p.i. Late-therapeutic i.v. 18 d.p.i	Tg4	No	CD62L^high^	Prevention Amelioration	^102^
PLP_139-151_ SJL/J	2 × 10^5^	Prophylactic i.v. 0 d.b.i.	GFP-Foxp3 x 5B6 TCR Tg	PLP_139–151_ (5 µM), TGF-β, RA, IL-2	CD62L^inter^ CD103^high^ CD73^+^ CTLA4^+^ GITR^+^	Prevention	^108^
C57BL/6 MOG_35–55_	1 × 10^5^	Late-therapeutic i.n. 15 d.p.i.	CAR-MOG	IL-2	n.a.	Prevention	^103^
C57BL/6 MOG^35–55^	1 × 10^6^	Therapeutic i.p. 10 d.p.i.	NO-Tregs	No	IL-10	Amelioration	^104^
MBP Ac1-9 Tg4	5 × 10^6^	Prophylactic i.p. 2–3 d.b.i.	Tg4	IL-2, TGF-β1	Helios^−^Eos^−^CD103^+^ GITR^+^NRP-1^+^ CD62L^+^ CTLA4 + IL-10	Prevention	^106^
MBP Ac1-9 (C57BL/6 × B10.PL)F1	1 × 10^6^	Prophylactic i.v. 1 d.b.i.	Tg4 Tbet^-^	No	CD62L^hi^	Prevention	^111^
MOG_35–55_ (Tg(HLA-DR15)#Lfug)	2 × 10^6^	Late-prohylactic i.v. 7 d.p.i	Ob2F3	MBP_85–99_, IL-2	Helios, GARP, and LAP	Amelioration	^112^
MOG_35–55_ or PLP_178–191_ C57BL/6	1 × 10^6^	Prophylactic i.v. 1 d.b.i. Therapeutic i.v. 9 d.p.i	MOG/NF-M TCR	IL-2, rapamycin	n.a.	Amelioration	^105^

*ATRA* all-trans retinoic acid, *CAR* chimeric antigen receptor, *CRP* C-reactive protein, *d.b.i.* days before immunization, *d.p.i.* days post immunization, *inh.* inhibition, *n.a.* not analyzed, *NF-M* neurofilament-medium, *o.e.* over-expression

^a^Prophylactic treatment: treatment before or at the moment of EAE induction; late-prophylactic treatment: treatment after EAE induction but prior to clinical onset and therapeutic treatment: first dose administrated to mice with the first clinical signs and late-therapeutic: treatment 5–8 days after the initial clinical signs

Stephens et al. administered Tg4 CD25^+^CD62L^hi^ MBP(Ac1-9)-reactive Tregs to Ac1-9 peptide-immunized B10.PL or B10.PL x SJL mice at 18 days post immunization (d.p.i.).^101^ This was the time of remission from the first EAE relapse. While mice that were not treated with Tregs developed more relapses and chronicity of EAE in the subsequent days, the severity of EAE relapse was markedly reduced in Treg-treated mice. The effect was more pronounced in B10.PL x SJL mice, as Treg-treated mice were nearly disease-free at the end of the observation period (100 d.p.i.). These results imply that Treg application could be efficient in MS, even at the later stages of the disease when the vicious cycle of autoimmune reactivity is well established. In another study, Fransson et al. used Tregs derived from CD4^+^ T cells that had been modified with a lentiviral vector system to express a chimeric antigen receptor (CAR) targeting MOG in trans with the FoxP3 gene. They were able to produce Tregs with a strong affinity for MOG and persistent FoxP3 expression.^102^ Tregs were applied to C57BL/6 mice with MOG_35–55_-induced EAE on day 15, i.e., at the time of the EAE peak. The treatment reduced the severity of EAE in the chronic phase, and the treated mice were symptom-free at the end of the observation period (30 d.p.i.). Importantly, Treg-treated mice were reimmunized with MOG_35–55_ at 30 d.p.i., and only one mouse developed any clinical manifestations of EAE. This effect on the clinical score occurred in parallel with inhibition of IL-12 and IFN-γ expression in the CNS. Interestingly, Tregs were applied to mice intranasally (i.n.), and the authors were able to demonstrate their migratory capacity (presumably via olfactory pathways), as they detected the Tregs within the CNS.

Niedbala et al. used nitric oxide (NO)-induced Tregs in EAE.^103^ They applied Tregs in C57BL/6 mice with MOG_35–55_-induced EAE at day 10, i.e., when the initial clinical signs appeared. The treatment led to a reduction in the severity of chronic EAE throughout the observation period, which ended at 30 d.p.i. This effect occurred in parallel with the limitation of immune cell infiltration into the CNS and a specific reduction in Th17 cell numbers within the CNS.

Malviya et al. generated transgenic T cells expressing a TCR specific for MOG and neurofilament medium (NF-MT) and used these T cells to treat C57BL/6 mice with EAE induced by MOG_35–55_ or PLP_178–191_.^104^ These engineered Tregs reduced the severity of EAE when applied at 9 d.p.i. (when clinical symptoms were evident), and their effect increased towards the peak of EAE. Importantly, these Tregs containing TCRs specific for MOG were equally efficient in EAE induced by MOG_35–55_ and EAE induced by an unrelated CNS antigen, PLP_178–191_. Such efficacy in restricting autoimmune reactivity against unrelated CNS antigens, if extrapolated to humans, would be a beneficial therapeutic property in MS. The engineered Tregs were detected in the CNS of the treated mice, and the authors suggested that the therapeutic effect of the Tregs was achieved within the CNS.

To summarize, at least in the EAE model of MS, a single application of Tregs to mice when the disease is well established is successful and has persistent effects. The cells can be applied systemically (i.v. or i.p.)^101,103–111^ or locally (i.n.).^102^ Nevertheless, the number of cells used for i.n. application was 10 times lower than that used for systemic application, which could be of interest when thinking about translation to humans. However, this difference in the number of Tregs required for efficient application could also be a consequence of different backgrounds and preparations. In conclusion, the results obtained in EAE clearly imply that the application of Tregs is a promising approach for the treatment of ongoing CNS autoimmunity, as observed in MS; therefore, clinical trials with Tregs in MS are expected to yield promising results.

#### Clinical trials in MS patients

Tregs, mainly tTregs, have been extensively tested in clinical trials for the treatment of not only AD and graft-versus-host disease after bone marrow transplantation as a prophylaxis for solid organ rejection but also for unexpected indications such as thalassemia, muscle dystrophies or amyotrophic lateral sclerosis.^92^ We recently accomplished a phase I/IIa trial of autologous CD4^+^CD25^hi^CD127-FoxP3^+^ Tregs administered to RRMS patients, either i.v. or intrathecally (i.t.) (trial registration: EudraCT: 2014-004320-22.^94^ The therapy proved to be safe. Although very preliminary, the results also suggested that intrathecal administration was more effective than intravenous administration. Experiments with adoptive transfer of Tregs suggested the good safety profile of Tregs administered via both tested routes.^94^

#### Dose, timing, and route of administration

To translate a treatment from early animal use into late human pathology, the dose and route of administration of the cells should be adjusted to address the level of inflammation and follow the progression of the disease. We tried to address these issues with the use of two routes of Treg administration. Patients treated i.v. received 40 × 10^6^ Tregs/kg b.w., which in our experience is a relatively high dose. Within this arm of the trial, we tried to address the hypothesis that systemic dysregulation between Tconvs and Tregs triggers the disease and relapse.^112^ The results suggest that we were, at least partially, ‘too late’, as half of the treated patients experienced relapse and progression of disease, as confirmed with MRI. This somewhat confirms that the clinical onset of disease may occur very late in the pathogenesis of MS, when the core of the process has already moved from the periphery to the CNS. The initiation of RRMS occurs somewhere in the peripheral lymphoid system with the presentation of myelin peptides and the generation of autoaggressive Tconvs, as in EAE, which is not adequately controlled by Tregs.^113^ However, Tconvs very quickly traffic to the CNS, destroy the BBB, attack myelin sheaths, and cause the development of lesions. Hence, the systemic administration of drugs is of limited value when symptoms have already occurred. The results of immunophenotyping in our trial seem to confirm such overactivity of Tconvs, which were mainly of an ‘experienced’ memory phenotype in all patients. At the same time, the majority of Tregs were naïve, confirming their relative inactivity. Surprisingly, MS patients exhibited an extraordinarily high percentage of peripheral Helios^−^FoxP3^+^ Tregs (20–30% of all FoxP3^+^ Tregs). These cells arise during the immune response, which suggests a history of long/massive immune activation in the periphery, with ineffective regulation of this process in MS. There have been reports that Tregs in RRMS patients follow autoreactive Tconvs and, attracted by inflammation, move quickly to the CNS, accumulating in the CSF. Moreover, remission occurs only when Tregs have accumulated in the CSF.^114^

This finding justifies the second arm of our trial, in which patients received Tregs i.t. The dose could be lower (1.0 × 10^6^ Tregs) in these patients, as 100% of the cells were delivered behind the BBB. The patients did not experience relapse, and MRI confirmed stable nonprogressing lesions in the CNS of these patients, which proved that this approach should be further tested in future trials.^94^

#### Novel Treg therapies

The approach based on engineered Tregs came from cancer studies in which the receptor specific for a particular molecule expressed on cancer cells was inserted into Tconvs using a vector (chimeric antigen receptor T (CAR-T) cells). This approach allows CAR-T cells to identify and kill cancer cells in a very specific and efficient way. Several drugs based on this therapy, i.e., axicabtagene ciloleucel (Yescarta, Gilead) and tisagenlecleucel (Kymriah, Novartis), are already routinely used. The first CAR-Tregs were constructed with specificity towards allo-HLA to quench the possible rejection of an allotransplant.^112,115,116^ The challenge is much higher in ADs such as MS, in which the target antigens are not as obvious. In the majority of such diseases, the complete list of autoantigens is not known.^117^ Moreover, it is possible that the target antigens evolve with the progression of the disease due to epitope spread.^114^ The complexity of the response is also caused by the fact that the same epitopes can trigger responses in both Tregs and Tconvs, and the final outcome depends on which subset prevails.^118^ Nevertheless, there have been attempts to create Tregs with engineered TCRs to direct them to particular sites and protect particular organs from autoimmune attack.^119^ These attempts for MS are at the EAE stage. There have been reports on CAR-T cells with specificity towards MOG manufactured from Tconvs and directed toward a regulatory function through *foxP3* gene delivery. This cellular product trafficked to the brain and exerted suppressive activity.^102^ More recently, human Tregs that had a transgenic TCR specific for MBP and had proven immunosuppressive activity were described.^111^

### Mesenchymal stem cells

MSCs are nonhematopoietic multipotent and self-renewing progenitor cells with the potential to differentiate into different lineages under specific conditions. They were described for the first time in 1968 by Friedenstein et al. as an adherent fibroblast-like population in the bone marrow that was able to differentiate into adipocytes, chondrocytes and osteocytes.^120^ In 1991, Caplan et al. named these cells “mesenchymal stem cells”.^121^ The authors demonstrated that MSCs are involved in bone and cartilage turnover and examined how surrounding conditions play a crucial role in their differentiation. Therefore, MSCs were postulated to be a novel therapeutic strategy for self-cell repair.^121^

MSCs can be isolated from various tissues, including the bone marrow, adipose tissue, placenta, umbilical cord, fetal liver, muscle, and lung. Among these tissues, bone marrow and adipose tissue are the most widely used sources of MSCs for therapeutic purposes. According to the Mesenchymal and Tissue Stem Cell Committee of the International Society for Cellular Therapies,^122^ the cells must be plastic adherent and able to differentiate into osteoblasts, adipocytes and chondroblasts, and their phenotype must be characterized by flow cytometry as CD105^+^ CD73^+^ CD90^+^ CD45^−^ CD34^−^ CD14^−^/CD11b^−^ CD79a^−^/CD19^−^ HLA-class II^−^. MSCs can be easily cultured and expanded ex vivo and have several properties, such as functions in tissue repair and homeostasis maintenance, immunomodulatory properties and low immunogenicity.

Due to their potential as an immunomodulatory and regenerative therapy, MSCs have been considered an optimal candidate cellular therapy for inflammatory and neurodegenerative diseases of the CNS, such as MS.

#### Preclinical studies

MSC-based cell therapy is the most investigated and has been examined in numerous in vitro and in vivo studies (EAE). In vivo treatment of EAE mice with bone marrow-derived MSCs (BM-MSCs) using several routes of administration (i.v., intraventricular (i.v.t.), and i.p.) has shown clinical amelioration of EAE severity, with reduced inflammatory infiltration, demyelination, and axonal damage. Of note, this beneficial effect was not found when BM-MSCs were infused during the chronic phase of the disease.^123^

It has been widely reported that MSC therapy in EAE exerts an important immunomodulatory effect and, to a lesser extent, a neuroprotective effect that results in axonal and neuronal protection through the release of antiapoptotic, antioxidant, and neurotrophic factors (systematically reviewed in^124^). Induction of Tregs, TGF-β1, and IL-10 mRNA in the spleen and lymph nodes of treated mice was the main immunological mechanism involved in the induction of peripheral tolerance following MSC transplantation in EAE mice.

#### Mechanisms of action of MSCs

The specific mechanisms that mediate the clinical benefits of BM-MSCs likely involve a combination of peripheral autoimmune modulation and the induction of CNS tissue protection. MSCs have four main properties: (1) migration capacity, (2) immunomodulation, (3) differentiation and neuroregeneration, and (4) secretion of soluble factors.

After their systemic administration, MSCs can migrate to and engraft in inflamed locations, exerting a local effect. Injured cells and immune cells involved in the immune response regulate MSC migration through the secretion of a broad range of signals, such as growth factors and chemokines. In vitro studies proved that MSC migration is regulated by receptors such as platelet-derived growth factor and insulin-like growth factor 1 and chemokine receptors such as CCR2, CCR3, CCR4, and CCL5.^125^ Studies in animal models have shown that MSCs can roll and tether to the endothelium, crossing the BBB through the VLA-4/VCAM-1 interaction. Moreover, MMPs play an important role in the transit of MSCs through the endothelial membrane. Interestingly, it is important to keep in mind that culture conditions during MSC ex vivo expansion can affect the expression of some receptors, such as VLA-4 and MMP, thus altering the migration capacity of MSCs.^125–127^

The immunomodulatory properties of MSCs can be exerted via cell-to-cell interactions or paracrine effects. On T cells, MSCs inhibit T cell proliferation via a mechanism independent of apoptosis induction.^128^ After coculture with T cells, MSCs decreased the Th1 response and induced a switch towards the Th2 response (a decrease in IFN-γ secretion and an increase in IL-4 secretion).^129^ In addition, MSCs induce the expansion of Treg subsets, increasing Foxp3 and CD25 expression. These Treg cells express TGF-β1 and PGE2.^129,130^

Regarding B cells, it has been reported that PDL1 inhibits their proliferation in murine cells and arrests the cell cycle in human cells.^131,132^ Chemotactic properties are also affected by soluble factors secreted by MSCs. A decrease in the expression of some chemokine receptors, such as CXCR4, CXCR5, and CCR7, was observed in B cells, together with a decrease in CXCL12 (a CXCR4 ligand) and CXCL13 (a CXCR5 ligand) expression in MSCs. In contrast, B cell costimulatory molecule expression and cytokine production were unaltered.^132^ Furthermore, suppression of B cell terminal differentiation by soluble factors secreted by MSCs, such as MCP-1 or IL-6, was also reported in C57BL/6 mice.^133^

MSCs induced the inhibition of NK cell proliferation when cocultured in IL-15-supplemented medium via both cell-to-cell contact and soluble factors, such as TGF-β1 and PGE2. Moreover, under these conditions, a decrease in the production of IFN-γ and IL-10, a decrease in the surface expression of CD56 in NK cells (although no changes were observed in the ratio of the CD56^dim^ and CD56^bright^ subsets), and lower cytotoxicity against HLA class I targets were found.^129,134,135^

MSCs can inhibit the maturation of DCs, resulting in a decrease in their capacity to activate alloreactive T cells. Furthermore, MSCs can inhibit TFN-α release by DCs, resulting in a tolerogenic state. Moreover, it has been postulated that PGE2 secreted by MSCs plays an important role in promoting the Th2 cell response, acting against Th1 cells, during the DC-induced Th cell response.^129,136^

As previously mentioned, MSCs release multiple soluble and encapsulated molecules, such as growth factors, cytokines, and chemokines (secretome), which can exert a biological effect in tissues. The conditioned medium of MSCs was shown to have a beneficial effect in damaged tissue in the liver and myocardium. Among these molecules are PGE2, which has anti-inflammatory and antiproliferative effects; IL-10 and IL-1 receptor antagonist (which have anti-inflammatory effects); and TGF-β1 and hepatocyte growth factor (which suppress T cell proliferation).^126,127^ Moreover, it was recently reported that exosomes released by MSCs can cross the BBB due to their small size and transfer bioactive molecules. In this regard, MSC-derived exosomes polarized microglial cells mainly into the M2 anti-inflammatory phenotype, promoting a shift to an anti-inflammatory profile and, consequently, reducing the clinical symptoms of EAE in rats.^137^

One important characteristic of MSCs is their ability to differentiate towards both mesenchymal and ectodermal cells, such as neurons, astrocytes, and oligodendrocytes. When analyzing the neuroprotective and regenerative functions of MSCs in MS, different experiments demonstrated that after i.v. administration of MSCs, they spread and homed preferentially to inflamed tissues, to the CNS in the case of EAE, to induce neuronal axon protection and the regeneration of damaged areas.^138,139^ Unfortunately, MSCs have not been demonstrated to transdifferentiate into neuronal cells. Their protective function is likely related to the secretion of antiapoptotic, anti-inflammatory, and neurotropic factors (through the activation of astroglial cells to secrete neurotrophins such as BDNF, glial cell-derived neurotrophic factor, and nerve growth factor),^139^ as well as the probable recruitment of local progenitor cells for subsequent differentiation into neurons and oligodendrocytes.^139–141^ These promising results encourage the use of MSCs in MS.

#### Clinical trials in MS

The number of clinical trials investigating MSC treatment for RRMS and progressive MS patients has increased quickly over the past two decades. Currently, more than twenty clinical trials are registered at ClinicalTrials.gov (see Table 4). Thus far, the results have shown that MSC therapy in MS patients is safe, with no relevant side effects.^142–151^ Due to variability in the protocols used (different types of MSCs, routes of administration, and doses) and the limited number of patients enrolled in each trial, most studies have been unable to draw conclusions about the efficacy of these treatments. Nevertheless, some trials have reported beneficial effects of MSCs through a decrease in relapse rate or disability.^143,146,147^

**Table 4:** Clinical trials using mesenchymal stem cells in MS patients

Source of cells	Years	Country	Phase	NCT number	Type of study	MS type	*n*	EDSS	Route of administ	Dose of MSCss	Number of administrations	Immunological findings	Ref
aMSCs	2013	Italy	I/II	NCT01854957	Randomized, double-blind, placebo-compared	Active PPMS, SPMS, RRMS	185	2.5–6.5	i.v.	A dose of 1–2 × 10^6^ MSCs/kg body weight	Single	Unreported	^142^
aMSCs	2013–2016	UK	I/II	NCT01606215									
aMSCs	2014–2018	Canada	II	NCT02239393									
aBM-MSCs	2015–2018	France	I/II	NCT02403947									
aBM-MSCs	2013–2016	Spain	I/II	NCT02035514									
aADMSCs	2012–2015	Sweden	I/II	NCT01730547									
aMSCs	2011–2018	Iran	I/II	NCT01377870	Randomized, double-blind, placebo-compared	RRMS, SPMS, RRMS	22	3–6.5	i.v.	Unreported	Single	Unreported	Unpublished
aMSCs	2010–2014	Spain	II	NCT01228266	Randomized, double-blind, placebo-controlled, crossover	RRMS	9	3–6.5	i.v	A dose of 1–2 × 10^6^ MSCs/kg body weight	Single	Non significative decrease of Th1 and Th17 cells; increase of Breg	^143^
aMSCs	2011–2016	USA	I	NCT00813969	Open-label	RRMS, SPMS	24	3–6.5	i.v.	A dose of 2 × 10^6^ MSCs/kg body weight	Single	Unreported	^144^
aBM-MSCs	2014–2016	Israel	II	NCT02166021	Randomized, double-blind, placebo-compared	PPMS, SPMS	48	3.5–6.5	i.t. and i.v.	10 × 10^6^/mL to 15 × 10^6^/mL	Double	Unreported	Unpublished
aBM-MSCs	2006–2011	UK	I/II	NCT00395200	Open-label	SPMS	10	2–6.5	i.v.	A dose of 2 × 10^6^ MSCs/kg body weight	Single	Unreported	^145,146^
aBM-MSCs	2013–2017	Jordan	II	NCT01895439	Open-label	MS	13	≤6	i.t.	Unreported	Unreported	Unreported	Unpublished
aBM-MSCs	2006–2009	Israel	I/II	NCT00781872	Open-safety clinical trial	Active MS and ALS	15 MS and 19 ALS	4–8	i.t. and i.v.	60 × 10^6^ i.t. (*n* = 34) and 25 × 10^6^ i.v. (*n* = 14)	Single	Increase of Treg (CD4+ CD25+) and decrease of CD83+ and CD86+ on DC and activated CD40+ cells. Decrease of in vitro lymphocytes proliferation to PHA	^147^
aBM-MSCs	2017	Jordan	I	NCT03069170	Open-label	RRMS	50	3–6.5	i.t. and i.v.	Unreported	Single	Recruiting IgG, IgA, and IgM levels and complement factors C3 and C4 will be analysed	Unpublished
aBM-MSCs	2015–2017	India	I/II	NCT02418351	Open-label	RRMS	69	3.5–6.5	i.t. and i.v.	50 × 10^6^ BM-MNC (i.v.) and 100 × 10^6^ BM-MNCs i.t. (when associated with CCSVI)	Single	Unreported	Unpublished
alloUCMSCs	2015–2017	Trinidad and Tobago	I/II	NCT02418325	Open-label	RRMS	69	3.5–6.5	i.t. and i.v.	50 × 10^6^ UC-MSCs (i.v.) and 100 × 10^6^ UC-MSCs i.t. (when associated with CCSVI)	Single	Unreported	Unpublished
aBM-MSCs	2015–2018	Spain	I/II	NCT02495766	Quadruple-blind, placebo-controlled study	RRMS and SPMS	8	<6.5	i.v.	Unreported	Single	Unreported	Unpublished
aBM-MSCs	2014–2018	UK	II	NCT01815632	Double-blind, placebo-controlled study	SPMS and PPMS	80	4–6	i.v.	500–600 mL bone marrow	single	Unreported	^148^
aBM-MSCs	2013–2018	UK	II	NCT01932593	Double-blind, placebo-controlled study	SPMS and PPMS	6	4–6	i.v.	500–600 mL bone marrow	Reinfusion (extension of NCT01815632)	Unreported	^149^
aADMSCs	2010–2015	Spain	I/II	NCT01056471	Triple-blind, placebo-controlled study	SPMS	30 [11 placebo, 10 low-dose and 9 high-dose]	5.5–9	i.v.	Low-dose (1 × 10^6^ cells/kg) or high-dose (4 × 10^6^ cells/kg)	Single	Unreported	^150^
aADMSCs	2014–2018	German	I	NCT02326935	Open-label	MS patients	2	Unreported	i.v.	150 × 10^6^ cells	Single	Unreported	Unpublished
aASVF	2014–2018	USA	II	NCT02157064	Open-label	MS patients	221	Unreported	Unreported	Unreported	Unreported	Unreported	Unpublished
alloUCMSCs	2014–2017	Panama	I/II	NCT02034188	Open-label	MS patients	20	2–5.5	i.v.	Infusions of 20 × 10^6^ UCMSCs over 7 days	7 doses	Unreported	^151^
alloUCMSCs	2010–2014	China	I/II	NCT01364246	Open-label	Progressive MS and NMO	20	Unreported	Unreported	Unreported	Unreported	Unreported	Unpublished
alloUCMSCs	2017	Jordan	I/II	NCT03326505	Randomized, single-blind	MS patients	60	≤7	i.t.	Unreported	Single	Unreported	Unpublished

*aADMSCs* autologous adipose-derived MSCs, *aASVF* autologous adipose stromal vascular fraction, *aBM-MSCs* autologous bone marrow-derived MSCs, *alloUCMSCs* allogenic umbilical cord-derived MSCs, *ALS* amyotrophic lateral sclerosis, *aMSCs* autologous MSCs, *i.v.* intravenous, *i.t.* intrathecal, *MS* multiple sclerosis, *MSCs* mesenchymal stem cells, *PPMS* primary-progressive MS, *RRMS* relapsing-remitting MS, *SPMS* secondary-progressive MS

To establish a consensus protocol for the use of MSCs for treating MS patients, in 2010, a group of experts created the International Mesenchymal Stem Cell Transplantation Study Group (IMSCTSG). As a result, a large multicenter randomized, double-blind, crossover phase I/II clinical trial was initiated to analyze the safety and efficacy of a single i.v. dose of autologous BM-derived MSCs (Mesenchymal StEm cells for Multiple Sclerosis: MESEMS trial; NCT02403947).^142^ The results of this trial have not yet been published.

Recently, Sarkar et al. reported relevant data about the MSC secretome that must be considered in the context of treatment with MSCs. These authors found that chronic inflammatory stress in MS patients limits MSC functionality by altering the MSC secretome.^152^ Moreover, other factors, such as aging and the in vitro expansion of MSCs, also exert detrimental effects on their functionality.^152^ Therefore, an in-depth analysis of the autologous MSC secretome should be considered as a quality control measure before administrating MSC therapy.

Regarding the effect of MSC treatment on the immune response, only a few clinical trials have performed immune monitoring to elucidate the mechanism of action of this therapy. Llufriu et al. reported a decrease (although it was not statistically significant) in Th1 and Th17 cells and an increase in Breg cells, as analyzed by the expression of IFN-γ, IL-17, and IL-10, respectively, using intracellular cytokine staining.^143^ Karussis et al. described an increase in Treg cells (identified as CD4^+^CD25^+^), together with a decrease in the expression of CD83 and CD86 in DCs and CD40 in activated cells. Moreover, they performed functional analysis of the T cell response, which showed a decrease in proliferation in response to phytohemagglutinin (PHA).^147^ Finally, a clinical trial of the administration of autologous BM-MSCs is currently ongoing in Jordan and will analyze the levels of IgG, IgA, and IgM and complement factors C3 and C4 in treated patients (NCT03069170).

### Hematopoietic stem-cell transplantation

Hematopoietic stem-cell transplantation (HSCT) was established for the treatment of hematological malignancies such as multiple myeloma and leukemias due to the capacity of hematopoietic stem cells to differentiate into all hematopoietic cell types.^153^ Surprisingly, treated patients who had concomitant ADs experienced amelioration of their clinical symptoms following HSCT.^154^ As a result, high-dose immunosuppression followed by autologous HSCT (aHSCT) has been investigated for patients with severe MS, with the rationale of this therapy based on a “reset” of the immune repertoire to eliminate autoreactive T and B cells; thus, subsequent aHSCT would allow reconstitution with the hope that a new and more self-tolerant immune system is developed.^155^

HSCT is carried out through different steps: mobilization, harvesting, ablative conditioning, and transplantation of aHSCs.^156^ First, HSCs are collected from the peripheral blood of patients after the mobilization of HSCs from bone marrow using treatment with granulocyte colony-stimulating factor (G-CSF) or GM-CSF with cyclophosphamide to prevent possible MS relapse or worsening of clinical symptoms as a result of G-CSF or GM-CSF administration.^156^ After 4 or 5 days, cells are harvested by leukapheresis and cryopreserved. Additionally, an HSC purification step is performed by CD34 positive selection to eliminate possible autoreactive lymphocytes. Then, the patient receives ablative conditioning to eradicate autoreactive cells. Different regimens of immune ablative conditioning are used based on the intensity of ablation using different chemotherapeutics and immunosuppressive drugs. High-intensity ablative regimens involve a high i.v. dose of immunosuppressive therapy and are associated with high toxicity and, in a small percentage of patients, mortality.^156,157^ In contrast, low-intensity regimens are nonmyeloablative and produce fewer adverse effects but may be associated with the early reappearance of MS disease activity post infusion. Hence, intermediate-intensity ablative regimens, referred to as BEAM (BCNU (or carmustine), etoposide, cytosine arabinoside, melphalan) or modified BEAM, are becoming more accepted.^157^ Following ablative conditioning, cryopreserved HSCs are thawed and reinfused into the patient.

Despite the promising positive results obtained using aHSCT (see below), some aHSCT-related risks must be considered: as stated above, the risk of transplant-related mortality, which is most severe in the first 100 days after transplantation, and the increased susceptibility to infection as a result of the accompanying chemotherapeutic immunosuppressive regimen. In addition, long-term side effects include the development of secondary autoimmune problems and/or fertility issues.^158^ Interestingly, aHSCT has dramatically improved over the years, showing a 0.3% treatment-related mortality rate since 2005.^157^

#### Clinical trials in MS

Since the end of the 1990s, several clinical trials evaluating the safety and efficacy of aHSCT using different conditioning regimens have been performed. Published results have shown that aHSCT can inhibit MS disease activity for 4–5 years in 70–80% of patients. Interestingly, this rate is higher than that achieved with any other therapy for MS. The results were better in young patients with inflammatory-active RRMS (reviewed in^157^).

To go one step further in examining aHSCT, currently, a phase III randomized clinical trial in RRMS patients with significant inflammatory disease activity is being conducted to compare the efficacy of aHSCT using a nonablative conditioning regimen with that of alemtuzumab (anti-CD52), which is considered the most effective available drug for RRMS (NCT03477500). Importantly, if the results indicate improved efficacy of aHSCT over alemtuzumab, aHSCT will likely be approved as a part of the current standard treatment recommendations for a significant proportion of RRMS patients (except in the case of Sweden, where aHSCT has been already approved). In the same way, another phase III clinical trial to analyze the efficacy of aHSCT (using high-dose myeloablative conditioning) in comparison with the best available therapy (BAT) in treatment-resistant RRMS is currently ongoing (BEAT-MS trial, NCT04047628). This multicenter, randomized, blinded study was conducted in a total of 156 RRMS patients distributed to each treatment arm at a ratio of 1:1. Treatments included in the BAT arm are natalizumab (anti-CD49d), alemtuzumab (anti-CD52), ocrelizumab (anti-CD20) and rituximab (anti-CD20). Relapse-free survival up to 3 years will be determined and used to compare the efficacy of aHSCT with that of the other treatments.

### T cell vaccination

Autologous T cell vaccination (TCV) involves collecting and expanding myelin-reactive T cells from MS patients and reinfusing them after their attenuation by irradiation. The rationale is that, as a result of this process, the immune system will attack pathogenic myelin-specific T cells, causing their deletion or inactivation while maintaining protective immunity.

The first study using attenuated MBP-reactive T lymphocytes was conducted in Lewis rats in 1981.^159^ The adoptive transfer of MBP-reactive T cells in rats induced EAE disease onset. Interestingly, administration of the same MBP-T cells attenuated by irradiation before adoptive induction resulted in disease prevention. Increased interest in TCV led to the initiation of multiple trials in MS patients in the late 1990s. The results from phase I pilot studies demonstrated that TCV treatment was safe and well tolerated^160–165^ (Table 5) and depleted MBP-reactive T cells after only 2 administrations.^160^ Interestingly, a correlation between MBP-reactive T cell depletion and a 40% reduction in the relapse rate was found in RRMS patients. Nevertheless, no relevant reduction in EDSS score was observed in the RRMS patients. In contrast, a slight increase in EDSS score after 2 years was reported in SPMS patients^166^ (Table 5).

**Table 5:** Clinical trials using T cell vaccination in MS patients

Vaccination type	Vaccine composition	NCT number	MS patients	Number of MS patients	Phase	Status	Study design	Dose and number of injections	Route of administration	Clinical outcome	TCV Center	Ref
T cell vaccination	Autologous MBP-reactive T cell clones (predominant reactivity pattern to MBP 84–102 and 143–168 peptides)	Unregistered	RRMS (*n* = 3) PPMS (*n* = 1) SPMS (*n* = 2)	6	Phase I	Completed	Non-randomized, open label	A total of 3 injections of 2–3 irradiated MBP-specific T cell clones (1 × 10^7^ to 1.5 × 10^7^ cells/clone)	s.c.	Decreased frequency of MBP-reactive T cells after the 2nd administration Remained undetectable in 5 of 6 patients at the end of the study	LUC, Diepenbeek, Belgium	^160^
T cell vaccination	Autologous MBP-reactive T cell clones	Unregistered	RRMS (*n* = 5) PPMS (*n* = 1) SPMS (*n* = 2)	8	Phase I	Completed	Non-randomized, open label	A total of 3 injections of 2–4 irradiated MBP-specific T cell clones (15 × 10^6^ cells/clone) every 2–4 months	s.c.	Moderate clinical improvement by reduction of relapse rate, and brain lesions compared to paired controls	LUC, Diepenbeek, Belgium	^161^
T cell vaccination	Autologous MBP-reactive T cell clones both whole MBP and MBP peptides (83–99 and 151–170)	Unregistered	RRMS (*n* = 28) SPMS (*n* = 26)	54	Phase II	Completed	Non-randomized, open label	A total of 3 injections every 2 months of 2–4 irradiated MBP-T cell clones (30 × 10^6^–60 × 10^6^ cells/clone)	s.c.	Reduction of 40% in ARR MRI lesions and EDDS remained stable.	Baylor College of Medicine, Houston, TX, USA	^166^
T cell vaccination	Autologous specific T-cell lines against MBP((83–99, 87–110, 151–170) and/or MOG(G (6–26, 34–56)	NCT00220428	RRMS with aggressive disease course and failure to respond to DMT	20	Phase I Phase II	Completed	Randomized, open label	A total of 3 injections in 6- to 8-week intervals of up to 1.5 × 10^7^ cells of each myelin-reactive T cell line (not exceeding 6 × 10^7^ total cells)	s.c.	Treatment was safe and no serious adverse events were observed. Decreased ARR, number and volume of active MRI lesions, as well as reduction in T2 lesion burden (quantitative MRI analysis)	Sheba Medical Center Ramat Gan, Israel	^169^
T cell vaccination	Autologous myelin-reactive T-cell lines against MBP, PLP, and MOG peptides	NCT00228228	Patients with probable MS within up to 3 months after the first clinical attack	80	Phase I Phase II	Unknown	Randomized, double-blind	Dose: 1–1.5 × 10^7^ cells/line, up to 5 lines Multiple injections: a total of 5 injections, 3 every 6 weeks and 2 boosts on weeks 24 and 28	Unknown	To evaluate the treatment effects at the onset of disease, in patients with probable MS within up to 3 months after the first clinical attack, in order to evaluate whether early treatment can prevent the second attack (conversion to definite MS).	Sheba Medical Center Ramat Gan, Israel	^165^
T cell vaccination	Whole bovine myelin selected T cells	Unregistered	SPMS (*n* = 4) with EDSS > = 6.5 SPMS (*n* = 80)	Phase I, *n* = 4 Phase II, *n* = 80	Phase I Phase II	Phase I completed	Non-randomized, open label	A total of 3 administrations every 3 months of 40 × 10^6^ irradiated T cells	s.c.	TCV using autologous irradiated bovine myelin-reactive T cells depletes circulating MBP-specific T cells in patients	USC, Los Angeles, USA	^162^
CSF-derived T cell vaccination	CSF-derived T cell lines reactive to myelin peptides	Unregistered	PPMS (*n* = 4)	4	Phase I	Completed	Non-randomized, open label	Single, twice or 3 administrations of 4 × 10^6^ irradiated CSF-derived T cell clones	s.c.	Administration of attenuated autoreactive CSF-derived T cell clones was feasible, and no adverse effects were observed	Harvard Medical School, Boston, USA	^168^
CSF-derived T cell vaccination	CD4+ T-cells derived from CSF	Unregistered	RRMS (*n* = 4) SPMS (*n* = 1)	5	Phase I	Completed	Non-randomized, open label	A total of 3 administrations every 2 months of 10 × 10^6^ irradiated CSF-derived T cells vaccines	s.c.	Vaccinations were well tolerated; no adverse effects were found. Preliminary efficacy data exhibited clinical stability of patients and reduced EDSS with no relapses during or after treatment	LUC, Diepenbeek, Belgium	^167^
T cell vaccination	T cells lines reactive against 9 myelin peptides of MBP (84–102, 143–168), MOG (1–22, 34–56, 64–86, 74–96) and PLP (41–58, 184–199, 190–209)	NCT01448252	Relapsing-progressive MS patients with EDSS from 3.0 to 7.0	26 MS patients: *n* = 19 patients treated with TCV and *n* = 7 patients treated with placebo	Phase I Phase II	Completed	Randomized double-blind, placebo-controlled	Dose: 10–30 × 10^6^ irradiated autologous T-cells Multiple administrations: 4 vaccinations on days 1, 30, 90, and 180	s.c.	Results demonstrate the feasibility and safety of the procedure and showed clinical improvements (decrease of EDSS and high frequency of relapse-free patients during the year of the study in TCV treated patients compared to placebo group) MRI parameters did not change significantly	Dept of Neurology, Hadassah Ein-Kerem Jerusalem, Israel	^163^
T cell vaccination Tcelna^®^ (Imilecleucel-T, previously known as Tovaxin^®^)	Autologous T cells against 6 immunodominant peptides derived from MOG (MOG1–17, MOG19–39), PLP (PLP30–49, PLP180–199) and MBP (MBP83–99, MBP151–170)	NCT00587691	RRMS (*n* = 9) SPMS (*n* = 7)	16	Phase I Phase II	Completed	Non-randomized, open label, dose-escalation study	Multiple administrations of 3 doses were tested. Low dose: 6–9 × 10^6^, mid dose: 30–45 × 10^6^ and high dose: 60–90 × 10^6^ irradiated autologous myelin-reactive T cells, administrated on weeks 0, 4, 12, and 20	s.c.	Tovaxin^®^ was safe and well tolerated, and appeared to be associated with clinical benefit (reduction of ARR) The administration of 30–45 × 10^6^ irradiated myelin-reactive T cells was the most effective dose during the 52 weeks study	Opexa Therapeutics, Inc. Texas, United States	^164^
T cell vaccination Tcelna® (Imilecleucel-T, previously known as Tovaxin®)	Autologous T cells against up to 6 immunodominant peptides derived from MBP, MOG, and PLP	NCT00245622 (TERMS study: Tovaxin for Early Relapsing Multiple Sclerosis)	RRMS (*n* = 95) CIS (*n* = 5)	150 (Tovaxin, *n* = 100; placebo, *n* = 50)	Phase IIb	Completed	Randomized, double-blind, placebo-controlled. Personalized therapy as a result of prescreening reactivity against peptide libraries covering MBP, MOG and PLP	Multiple administrations: 5 injections of 30–45 × 10^6^ irradiated autologous myelin-reactive T cells on weeks 0, 4, 8, 12, and 24	s.c.	Tovaxin treatment had a safety profile but efficacy results were not clear likely due to the presence of patients with prior treatment with DMT therapies	Opexa Therapeutics, Inc. Texas, United States	^170^
T cell vaccination Tcelna® (Imilecleucel-T, previously known as Tovaxin®)	Autologous T cells against up to 6 immunodominant peptides derived from MBP, MOG, and PLP	NCT00595920 OLTERMS study: Open label extension of TERMS study	RRMS CIS with positive myelin-reactive T cells (MRTC) in their blood during the previous TERMS study	116	Phase II (extension study)	Terminated due to financial constraints	Multicenter, randomized, double-blind, placebo-controlled. Personalized therapy using prescreening reactivity test	Multiple administrations: 5 injections of 30–45 × 10^6^ irradiated autologous myelin-reactive T cells on weeks 0, 4, 8, 12, and 24. yearly as required	s.c.	Since only 38 patients received the treatment and 32 of them did not complete the 5 administrations, it was not possible to evaluate the efficacy	Opexa Therapeutics, Inc. Texas, United States	–
T cell vaccination Tcelna® (Imilecleucel-T, previously known as Tovaxin®)	Autologous pool of myelin-reactive T-cells against immunodominant epitopes selected derived from MBP, MOG and PLP on a per subject basis	NCT01684761 Abili-T study	SPMS Presence of myelin-reactive T-cells EDSS score 3.0–6.0, inclusively	183	Phase II	Completed	Multicenter, randomized, double-blind, placebo-controlled	Multiple administrations: 5 injections of 30–45 × 10^6^ irradiated autologous myelin-reactive T cells on weeks 0, 4, 8, 12, and 24.	s.c.	Favorable safety and tolerability profile in SPMS patients No changes on disease progression rate nor reduction of brain atrophy was observed	Opexa Therapeutics, Inc. Texas, United States	–

*ARR* annualized relapse rate, *DMT* disease-modifying treatments, *i.d.* intradermal IFA, incomplete Freund’s adjuvant, *MBP* myelin basic protein, *MS* multiple sclerosis, *MOG* myelin oligodendrocyte glycoprotein, *PLP* proteolipid protein, *PPMS* primary-progressive MS, *RRMS* relapsing-remitting MS, *s.c.* subcutaneous, *SPMS* secondary-progressive MS, *TCV* T cell vaccination, *WBC* white blood cell counts

To improve TCV outcomes in MS patients, T lymphocytes isolated from CSF were used to develop CSF-derived T cell lines against myelin peptides, since CSF was thought to contain infiltrating pathogenic lymphocytes relevant to the disease process due to the proximity of these lymphocytes to the CNS. Data from two pilot studies indicated that the administration of attenuated autoreactive CSF-derived T cell clones was feasible and safe, and no adverse effects were observed. Phase II studies with a large number of MS patients are required to evaluate the clinical and radiological efficacy of CSF-derived TCV.^167,168^

To improve clinical remission, a new strategy using multivalent TCV was developed using immune-dominant epitope sequences of MBP, MOG and PLP. A phase I clinical trial was conducted by Achiron et al. using attenuated T cell lines specific for different MBP and/or MOG peptides.^169^ The results of the trial showed clinical as well as radiological benefits with no adverse effects in RRMS patients who did not respond to disease-modifying treatments.^169^ In addition, multivalent TCV allows personalized therapy following prescreening for myelin reactivity. In this regard, Tcelna (Imilecleucel-T, previously known as Tovaxin), a TCV composed of autologous preselected T cells reactive against up to six immunodominant peptides derived from MBP, MOG and PLP, was shown to be safe and well tolerated in a phase II clinical trial; however, its clinical and radiological efficacy was not demonstrated (TERMS trial: Tovaxin for Early Relapsing Multiple Sclerosis, NCT00245622,^170^). An extension study (OLTERMS trial: Open label extension of TERMS study, NCT00595920) was initiated to further evaluate the clinical efficacy of Tcelna/Tovaxin. However, the study was terminated because it did not accomplish the predefined primary endpoint of reducing brain atrophy or the secondary endpoint of decreasing disability progression (NCT00595920).

## Combined therapies

Evidence from phase I clinical trials conducted in type 1 diabetes, rheumatoid arthritis, Crohn’s disease and MS patients has demonstrated that tolDCs, Tregs, TCV, MSCs, and HSCT are safe and well tolerated. However, due to the different cell types and mechanisms involved in the maintenance of immune tolerance and the difficulty of establishing an optimal dose, route, and frequency of administration, it has been postulated that a combined therapy of antigen-specific cells with conventional immunomodulatory drugs is most likely necessary to potentiate their beneficial effects and restore immune homeostasis.^54^

### TolDCs with immunomodulatory/immunosuppressive drugs

A single infusion of donor-derived VitD3+IL-10-tolDCs before transplantation in combination with CD28 costimulatory signal blockade using the fusion protein CTLA-4 Ig (abatacept) and rapamycin significantly prolonged allograft survival in nonhuman primates by attenuating donor‐reactive memory T cells.^171^

More recently, the results of the ONE Study, an analysis of seven non-randomized, single-arm, phase I/IIa trials in living donor kidney transplantation in which different types of regulatory cells, including tolDCs, were combined with conventional immunosuppressive drugs reducing the proliferative response of lymphocytes by inhibiting IL-2 signaling (basiliximab and tacrolimus), blocking de novo synthesis of guanosine nucleotides (mycophenolate mofetil) or using a feedback mechanism to control inflammation and the immune response to steroids. Data from the trial showed the safety and feasibility of combined therapy. Interestingly, a decrease in infectious complications was reported in the group administered combined therapy.^100^ In this context and to move one step forward in the clinical application of tolDCs in MS, our group investigated the effect of tolDCs combined with IFN-β. We found that the anti-inflammatory and immunomodulatory properties of IFN-β combined with VitD3-tolDCs induced a reduction in Th1 and Th17 cells, favoring a more potent antigen-specific regulatory effect of VitD3-tolDCs both in vivo (EAE model) and in vitro in cultures of peripheral blood cells from MS patients (Quirant-Sánchez et al. unpublished data).

### Tregs with immunomodulatory/immunosuppressive drugs

The relationship between Tregs and IL-2 is probably the most explored area in MS research, with the longest history of clinical trials.^172^ Tregs have high expression of IL-2R and are highly dependent on IL-2; therefore, this cytokine or its muteins have been used alone or in combination with Tregs in many conditions, such as GvHD, transplantation or T1D.^96,97^ Other approaches include the administration of rapamycin as an immunosuppressive agent able to induce Tregs and tolDCs^98,99^ or adjuvant therapy with Tregs added to standard immunosuppression in organ transplantation.^100^ Our team has been testing Tregs combined with anti-CD20 antibody in T1D.^173^

### TolDCs in combination with Tregs

The combination of tolDC and Treg cell therapy has been proposed for the treatment of ADs since these cells can interact to stabilize, maintain and potentiate their tolerogenic effects.^174^ Although coadministration or serial administration of antigen-specific tolDCs and Tregs a priori does not seem to be feasible, a single leukapheresis could provide enough monocytes to generate tolDCs and lymphocytes for autologous Treg expansion.^174^ However, to date, no studies investigating the efficacy of this combined therapy have been reported.

### TolDCs/Tregs in combination with MSCs

A different combination therapy approach for ADs is the administration of antigen-specific cells, such as tolDCs or Tregs, with MSCs. In this context, synergistic suppression of autoimmune arthritis was reported when collagen-induced arthritis (CIA) mice were treated with RelB-silenced tolDCs and MSCs. Combined therapy was able to inhibit disease progression, decrease the clinical symptoms of CIA and reduce joint damage. Immunological studies revealed inhibition of the collagen T cell response and a shift towards an anti-inflammatory profile, although the most potent synergistic effect elicited by RelB-silenced tolDC and MSC therapy was a strong reduction in Th17 cells.^175^

Another example was published by Lee et al., who used a murine model of acute GVD (aGVHD) to determine the efficacy of combined Treg and MSC therapy.^176^ Researchers have shown that the adoptive transfer of donor-derived MSCs and Tregs reduces the severity of aGVHD by controlling Th1 and Th17 responses (related to the function of Tregs), accompanied by increased long-term survival of transferred Tregs and induction of endogenous Treg repopulation in target organs (related to MSC function).^176^

In conclusion, all these data suggest that combination therapies have the advantage of increasing the possible clinical effectiveness of antigen-specific cell-based tolerogenic therapies and will contribute to their optimal application in the future.

## Legal rules for cellular products

Cellular therapies are classified as either transplants, in which unmodified cells are immediately administered, or drugs, in which substantial laboratory modification of the cells and/or nonhomologous use of the cells occurs. In Europe, the use of these two kinds of products is regulated by directive 2004/23/EC, which sets standards of quality and safety for the donation, procurement, testing, processing, preservation, storage and distribution of human tissues and cells, and directive 1394/2007, which is focused on advanced therapy medicinal products (ATMPs). Clinical trials to test new investigational medicines are also centrally regulated by regulation 536/2014. In general, these directives implement the guidelines established by the International Council for Harmonization of Technical Requirements for Pharmaceuticals for Human Use (ICH), a common worldwide effort of national regulatory authorities and the pharmaceutical industry to discuss scientific and technical aspects of pharmaceuticals. Similar acts based on ICH rules are also in place in other parts of the world and describe the path of new medicines from discovery to routine use. This path for cellular drugs is similar to that for other drugs. It starts from preclinical assessment, which establishes the most important toxic, pharmacokinetic and pharmacokinetic features of the investigational drug. This step in humans consists of three trials: a first-in-man phase I trial to establish safety and dose, a phase II trial to test efficacy and a phase III trial to confirm safety and efficacy in a larger population. The last study is usually pivotal and the basis for marketing authorization. Drugs are also continuously surveyed post authorization, which is phase IV. In Europe, the authorization of cellular drugs is centralized, as it occurs in one step for all EU countries and is granted by the EMA. Some possibilities for early access to cellular products, such as hospital exemption rules, exist, but these are very limited, and there is pressure to provide standardized, equal treatment to all citizens of the EU. The importance of cellular drugs and high expectations that these cellular drugs will be a ‘game changer’ for many unmet medical needs are highlighted by the fact that there is a dedicated legal board within the EMA, the Committee for Advanced Therapies (CAT), which addresses only regulations on cellular therapy. There are also academic initiatives to regulate the way cellular drugs are manufactured. For example, academic guidelines for tolerance-inducing cellular products based on the minimal information model exist.^177,178^

## Concluding remarks

Over the past 20 years, an extraordinary effort has been made to develop treatments that can halt the natural evolution of MS. Thanks to this effort, we have a variety of drugs that, due to their powerful anti-inflammatory/immunosuppressive effects, decrease the rate of relapse and radiological activity, thus slowing the onset of disability. However, by acting in an immunologically nonspecific manner and suppressing one or more branches of the immune response, these treatments have the potential to cause serious adverse effects. In this context, cell therapy appears to be a promising strategy.

Phase I clinical trials with tolDCs, MSCs and Tregs in MS patients have shown these therapies to be safe and well tolerated, with no relevant adverse effects. Among cell-based strategies, MSCs have a potent immunomodulatory effect. TolDCs loaded with self-antigens against which tolerance is induced, Tregs, and more recently CAR-Tregs have the potential to specifically act against the cause of the disease, i.e., the autoimmune response to CNS myelin, while maintaining protective immunity. In addition, although less explored, some cell therapies show neuroprotective and neurorepair potential. These cells have shown promising results in experimental models, and some, such as hfNSCs, MSC-NPs and hESCs, are already being tested in patients.

All these developments paint a very hopeful picture for the next few years. The personalized combination of treatments will allow us to approach this disease from various fronts and, without a doubt, increase the possibility of obtaining a suitable therapy for each patient at the right time to ensure the highest quality of life possible because each patient is unique, as is their disease.
